# Differentiated T Lymphocytes and Cancer Cell Mitochondrial Metabolism to Enhance Radioimmunotherapy by a Biomimetic Nanozyme System

**DOI:** 10.1002/advs.202515097

**Published:** 2025-11-03

**Authors:** Hanyu Zhang, Yuhan Deng, Yantong Lu, Miao Wang, Kun Qiao, Zifan Yang, Shipeng Ning, Tong Liu

**Affiliations:** ^1^ Department of Oncology Surgery Harbin Medical University Cancer Hospital Harbin 150000 China; ^2^ Digestive Diseases Center The Seventh Affiliated Hospital Sun Yat‐sen University Shenzhen 518107 China; ^3^ Department of Breast Surgery The Second Affiliated Hospital of Guangxi Medical University Nanning 530000 China; ^4^ Research Center of Nanomedicine Technology The Second Affiliated Hospital of Guangxi Medical University Nanning 530000 China; ^5^ NHC Key Laboratory of Cell Transplantation Harbin Medical University Harbin 150081 China

**Keywords:** biomimetic nanozyme, mitochondrial metabolism, PKM2 activator, radioimmunotherapy, sustaining ROS production

## Abstract

Strategies to enhance the anti‐tumor immune response through the regulation of cellular metabolism are under intensive investigation. Herein, a T cell membrane (TCM)‐coated biomimetic magnesium carbonate (MgCO_3_)/Fe‐CD hybrid nanozyme system loaded with the Pyruvate kinase M2 (PKM2) activator TEPP‐46 (TFMP) is developed, designed to simultaneously induce mitochondrial metabolic reprogramming in both T cells and tumor cells following radiotherapy (RT). The TCM coating enables TFMP to specifically target tumor tissues that highly express PD‐L1, where it competitively binds to PD‐L1 and thereby alleviates immune checkpoint‐mediated T cell suppression. Upon X‐ray irradiation, TFMP continuously catalyzes the conversion of radiotherapy‐generated hydrogen peroxide into hydroxyl radicals, thereby sustaining reactive oxygen species production, which leads to mitochondrial damage and immunogenic cell death in tumor cells. Moreover, TFMP can neutralize the acidic tumor microenvironment, while the released Mg^2+^ and TEPP‐46 further augment T cell activation and mitochondrial function, thereby increasing the production of ATP and granzyme B, which effectively eliminate residual tumor cells. Experimental results demonstrate that the combination of TFMP and RT can significantly inhibit tumor progression and activate anti‐tumor immunotherapy. This TFMP enhances the efficacy of breast cancer radioimmunotherapy, offering a foundation for developing more comprehensive therapeutic approaches of breast cancer to achieve clinical benefits.

## Graphical abstract

T cell membrane‐coated MgCO_3_/Fe‐CD nanozyme loaded with TEPP‐46 (TFMP) enhances breast cancer radioimmunotherapy by reprogramming mitochondrial metabolism in T cells and tumors. It targets PD‐L1, converts H_2_O_2_ into ROS, neutralizes acidity, and releases Mg^2^⁺/TEPP‐46 to boost T cell activation. Combined with RT, it induces immunogenic cell death, inhibits tumor progression, and improves breast cancer treatment efficacy.

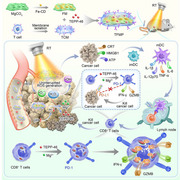

## 1 Introduction

Cancer remains a thorny problem.^[^ ^1^, ^2^, ^3^, ^4^, ^5^, ^6^ ^]^ Cancer immunotherapy, particularly PD‐1/PD‐L1 antibody therapy, has shown significant clinical efficacy.^[^ ^7^, ^8^ ^]^ Nevertheless, the overall response rate remains below 30%, and its therapeutic effectiveness is notably limited in certain types of solid tumors and lymphomas.^[^ ^9^, ^10^ ^]^ Signals originating from the tumor microenvironment (TME) contribute to immune cell exhaustion through mechanisms such as nutrient depletion and mitochondrial inhibition.^[^ ^11^, ^12^ ^]^ Mitochondria perform essential functions including oxidative phosphorylation (OXPHOS), calcium buffering, reactive oxygen species (ROS) production, metabolic regulation, and cytotoxic activity.^[^ ^13^ ^]^ The structural integrity and metabolic reprogramming of mitochondria are critical for the proper functioning of both T cells and tumor cells, influencing T cell differentiation from the naive to the effector state and simultaneously supporting tumor progression.^[^ ^14^ ^]^ Emerging evidence indicates that cancer cells can transfer mutated and dysfunctional mitochondria to T cells via tunneling nanotubes and extracellular vesicles.^[^ ^15^ ^]^ Ultimately, the incorporation of cancer‐derived dysfunctional mitochondria into T cells leads to the displacement of healthy mitochondria, impairing T cell activation and enabling tumor cells to evade immune surveillance. Therefore, simultaneously modulating mitochondrial metabolism in both T cells and tumor cells represents a promising strategy to enhance the efficacy of cancer immunotherapy.

To induce mitochondrial metabolism damage in tumor cells, we have thought of using radiotherapy (RT) to achieve this. As a conventional anti‐tumor strategy, RT primarily induces tumor cell death through two mechanisms: direct damage to critical biomolecules such as DNA and mitochondria, and indirect damage mediated by ROS via oxidative stress.^[^ ^16^, ^17^ ^]^ However, the ROS generated by RT alone is often insufficient, and intracellular antioxidants like glutathione (GSH) can neutralize ROS, leading to radioresistance.^[^ ^18^, ^19^, ^20^, ^21^ ^]^ Therefore, sustained induction of ROS following RT remains a critical challenge that needs to be urgently addressed. Nanozymes, owing to their enzyme‐mimicking catalytic properties, have demonstrated significant potential for clinical applications in the treatment of diseases including cancer.^[^ ^22^, ^23^, ^24^, ^25^, ^26^, ^27^, ^28^, ^29^, ^30^ ^]^ Compared with natural enzymes, nanozymes exhibit superior stability and reusability in complex physiological environments, overcoming the limitations imposed by the sensitivity of native enzymes to pH, temperature, and ionic strength.^[^ ^31^, ^32^, ^33^ ^]^ In recent years, nitrogen‐doped carbon‐based nanozymes have garnered considerable attention in the biomedical field due to their excellent photothermal properties and catalytic activity.^[^ ^34^, ^35^ ^]^ Among these, carbon dots (CDs), a class of 0D carbon‐based nanomaterials with sizes below 10 nm, have shown promising potential in anti‐tumor therapy, attributed to their outstanding optical properties, catalytic efficiency, and biocompatibility.^[^ ^36^ ^]^ Recent studies have demonstrated that in peroxidase‐like (POD‐like) catalytic reactions, iron‐doped carbon‐based nanozymes exhibit a higher utilization efficiency of active sites and can more effectively catalyze the decomposition of hydrogen peroxide (H_2_O_2_) to produce hydroxyl radicals (·OH).^[^ ^37^ ^]^ Therefore, the integration of carbon‐based nanozymes into a chemically cascading system to amplify ROS generation post‐RT may represent a promising strategy for enhancing RT sensitivity and mitochondrial metabolism damage.

Pyruvate kinase M2 (PKM2) catalyzes the conversion of phosphoenolpyruvate to pyruvate and carries out metabolic activities.^[^ ^38^ ^]^ PKM2 exists in transcriptionally active dimeric (di‐PKM2) or enzymatically active tetrameric form (tet‐PKM2).^[^ ^39^ ^]^ In addition to its metabolic functions, PKM2 also plays a regulatory role in CD8^+^ T cells immune responses. Recent multi‐omics studies have uncovered how PKM2 activation influences the transcriptome, epigenome, metabolome, and effector functions of CD8^+^ T cells.^[^ ^40^ ^]^ These findings demonstrate that PKM2 activation enhances CD8^+^ T cell activation and functional performance. Specifically, di‐PKM2 reprograms both the cellular metabolome and epigenome, leading to increased mitochondrial translation. Moreover, CD8^+^ T cells and CAR‐T cells with activated PKM2 exhibit enhanced memory recall responses and improved anti‐tumor efficacy following adoptive cell therapy. Therefore, combining RT sensitization with T cell PKM2 activation may synergistically modulate mitochondrial metabolism in both tumor cells and immune cells, thereby strengthening anti‐tumor immunity. However, research in this area remains limited, and further studies are needed to explore this potentially transformative therapeutic strategy.

**Scheme 1:**
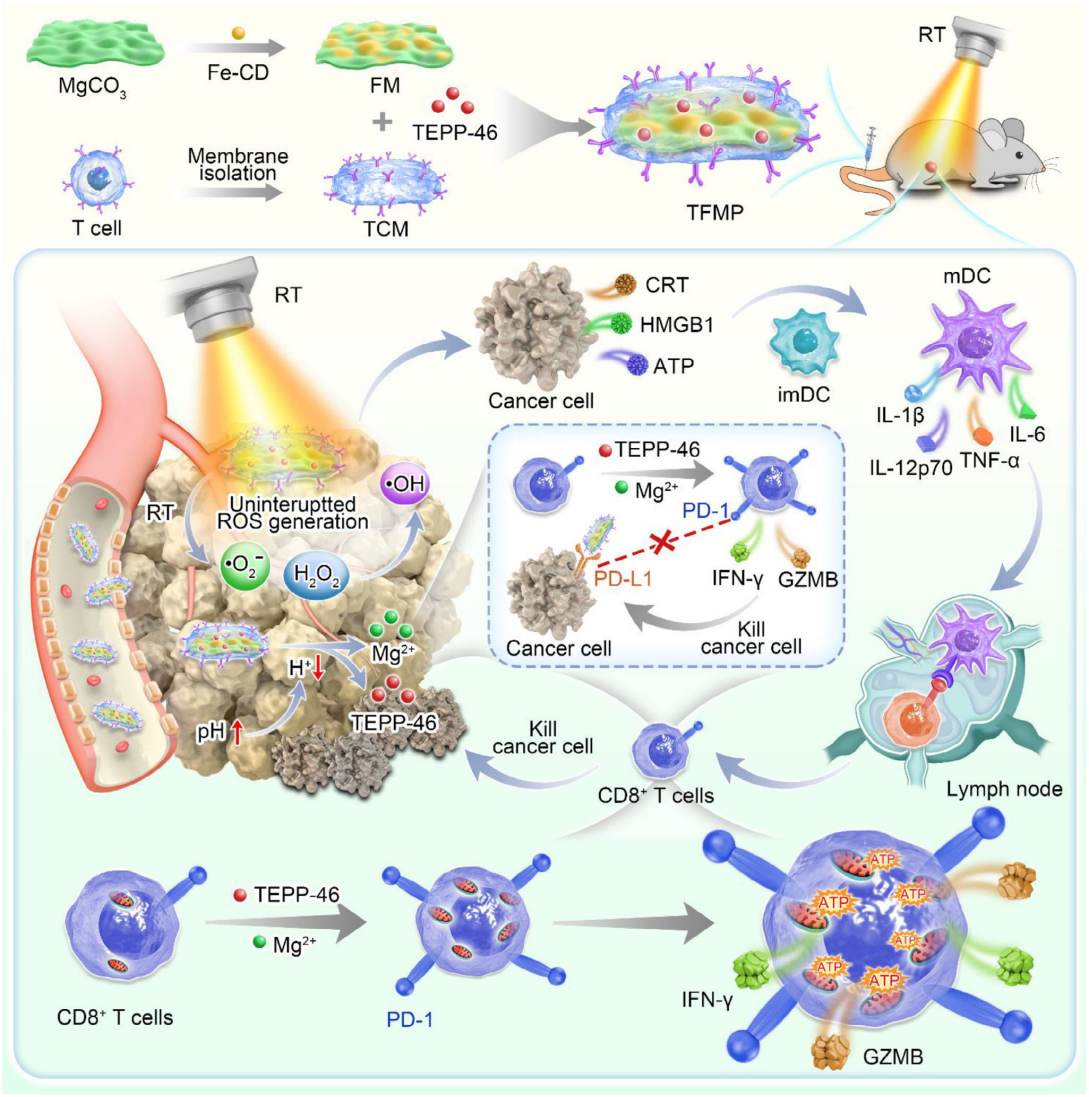
Schematic illustration of differentiated T lymphocytes and cancer cell mitochondrial metabolism to enhance radioimmunotherapy by a biomimetic nanozyme system.

Herein, we developed a T lymphocyte membrane‐biomimetic magnesium carbonate (MgCO_3_)/Fe‐CD hybrid nanozyme system loaded with PKM2 activator TEPP‐46, designed to simultaneously induce mitochondrial metabolic reprogramming in both T cells and tumor cells following RT (**Scheme** **1**). Following intravenous administration, TFMP specifically targeted PD‐L1‐overexpressing tumor cells and tissues. Subsequently, TFMP nanozyme catalyzes the hydrogen peroxide produced by RT, leading to sustained ·OH production and continuous ROS amplification. The combination of TFMP and RT induced mitochondrial dysfunction and immunogenic cell death (ICD) in tumor cells, resulting in the release of damage‐associated molecular patterns (DAMPs), which facilitated dendritic cell (DCs) maturation and T cell infiltration. The acid‐neutralizing reaction between MgCO_3_ and hydrogen ions alleviated the acidic tumor microenvironment. The released magnesium ions and TEPP‐46 further enhanced T cell activation and mitochondrial function, promoting ATP and granzyme (GZMB) production, thereby effectively eliminating residual tumor cells. Moreover, TFMP is competitively bound to PD‐L1, thereby alleviating immune checkpoint‐mediated suppression of T cells. Experimental results demonstrated that this nanozyme system achieved mitochondrial metabolic reprogramming in both T cells and tumor cells through multiple synergistic mechanisms. It effectively reversed the mitochondrial damage and metabolic suppression imposed by cancer cells on T cells, enhanced systemic antitumor immunity after RT, and significantly inhibited tumor metastasis. Compared to previously reported nanozyme‐based radioimmunotherapy systems,^[^ ^41^, ^42^ ^]^ TFMP places greater emphasis on dual regulation of tumor cell and T cell functions to achieve immune enhancement, demonstrating superior advantages in radioimmunotherapy.

## 2 Results and Discussion

### 2.1 Preparation and Characterization of FM

**Figure 1:**
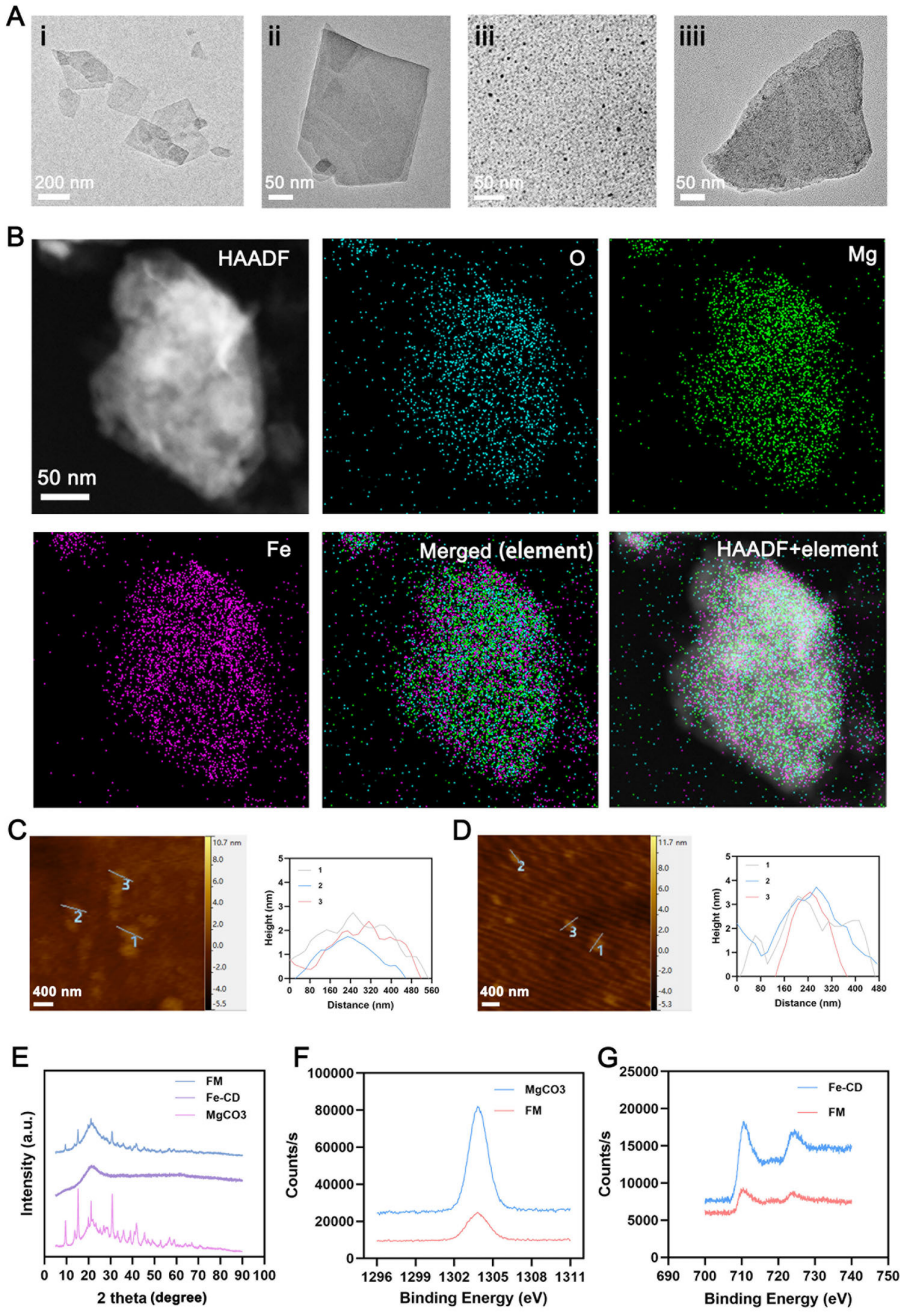
A) TEM image of MgCO_3_ nanosheets (i and ii), Fe‐CD (iii) and FM (iiii). B) HAADF‐SETM images and element mapping of FM. C) AFM image of the MgCO_3_ nanosheet and height profiles along the dashed lines in the AFM image.(D) AFM image of the FM and height profiles along the dashed lines in the AFM image. E) X‐ray diffraction pattern of FM, MgCO_3,_ and Fe‐CD. F) X‐ray photoelectron spectroscopy (XPS) spectrum of Mg and G) Fe for indicated formulations.

MgCO_3_ nanosheets were used in this work. Then, using hydrochloric dopamine and o‐phenylenediamine as carbon sources and FeCl_3_·6H_2_O as the dopant, iron‐doped carbon dots (Fe‐CDs) were synthesized through a simple one‐pot hydrothermal method.^[^ ^37^ ^]^ The Fe‐CDs can spontaneously adsorb onto the surface of MgCO_3_, and further be prepared into MgCO_3_ nanosheets loaded with Fe‐CDs, named FM. The morphology and size of MgCO_3_, Fe‐CDs, and FM were observed by transmission electron microscopy (TEM). As shown in **Figure** **1**A, MgCO_3_ is in a sheet‐like form, with uniform size and good dispersion, and sharp edges. Fe‐CDs are spherical and well‐dispersed. The TEM image of FM further confirmed the successful loading of Fe‐CDs onto MgCO_3_. High‐angle annular dark‐field scanning TEM (HAADF‐STEM) images and corresponding elemental maps taken on FM further confirmed the successful immobilization of Fe‐CDs on MgCO_3_ (Figure 1B). The atomic force microscopy (AFM) images and AFM height profiles of MgCO_3_ nanosheets and FM showed that MgCO_3_ nanosheets and FM are nanoscale, with an average thickness of ≈3 nm (Figure 1C,D). X‐ray diffraction tests were used to characterize the phase structure. As shown in Figure 1E, the characteristic peaks in FM nanosheets are highly consistent with the MgCO_3_ and Fe‐CDs, indicating that the crystal phase of MgCO_3_ nanosheets is well‐preserved after adsorbing Fe‐CDs. To further investigate the valence state and element distribution in FM, X‐ray photoelectron spectroscopy (XPS) analysis was conducted. The XPS results showed characteristic peaks of essential elements, especially Mg and Fe, indicating that Fe‐CDs were successfully integrated onto MgCO_3_ (Figure 1F,G). These results indicate the successful preparation of FM nanosheets. The Fourier Transform Infrared Spectroscopy (FTIR) spectra of Fe‐CD, MgCO_3_, and FM was shown in Figure S1 (Supporting Information). Near 1650 cm^−1^, in Fe‐CD, there is a stretching vibration peak of C = O, which originates from the carbonyl group in the carbon quantum dot. This group also appeared at a similar position in FM, but the peak position underwent a blue shift, and the peak intensity decreased. It is indicated that after Fe‐CD couples with MgCO_3_, the hydrogen bond is broken, a certain reaction occurs, and a new structure is produced. It is not a simple physical mixture. A stretching vibration peak of C‐O‐C was observed in the Fe‐CD sample near 1100 cm^−1^, corresponding to the ether bond in the sample. After coupling, the ether bond was retained, and the peak intensity increased, indicating that the reaction in the system was relatively mild and did not damage the structure of the ether bond. At the same time, it increased the ordering of the ether bond, which was macroscopically manifested as an increase in peak intensity. At the 750 cm^−1^ positions, the stretching vibration peak of Fe‐O can be observed in Fe‐CD, and this peak is also reflected in FM. This peak mainly results from the coordination between iron ions and oxygen‐containing groups in carbon, indicating that Fe elements can still coordinate well after coupling with MgCO_3_, which assists in proving successful coupling. The Energy Dispersive Spectrometry (EDS) analysis proved that FM contains iron and magnesium elements (Figure S2, Supporting Information).

### 2.2 Preparation and Characterization of TFMP

**Figure 2:**
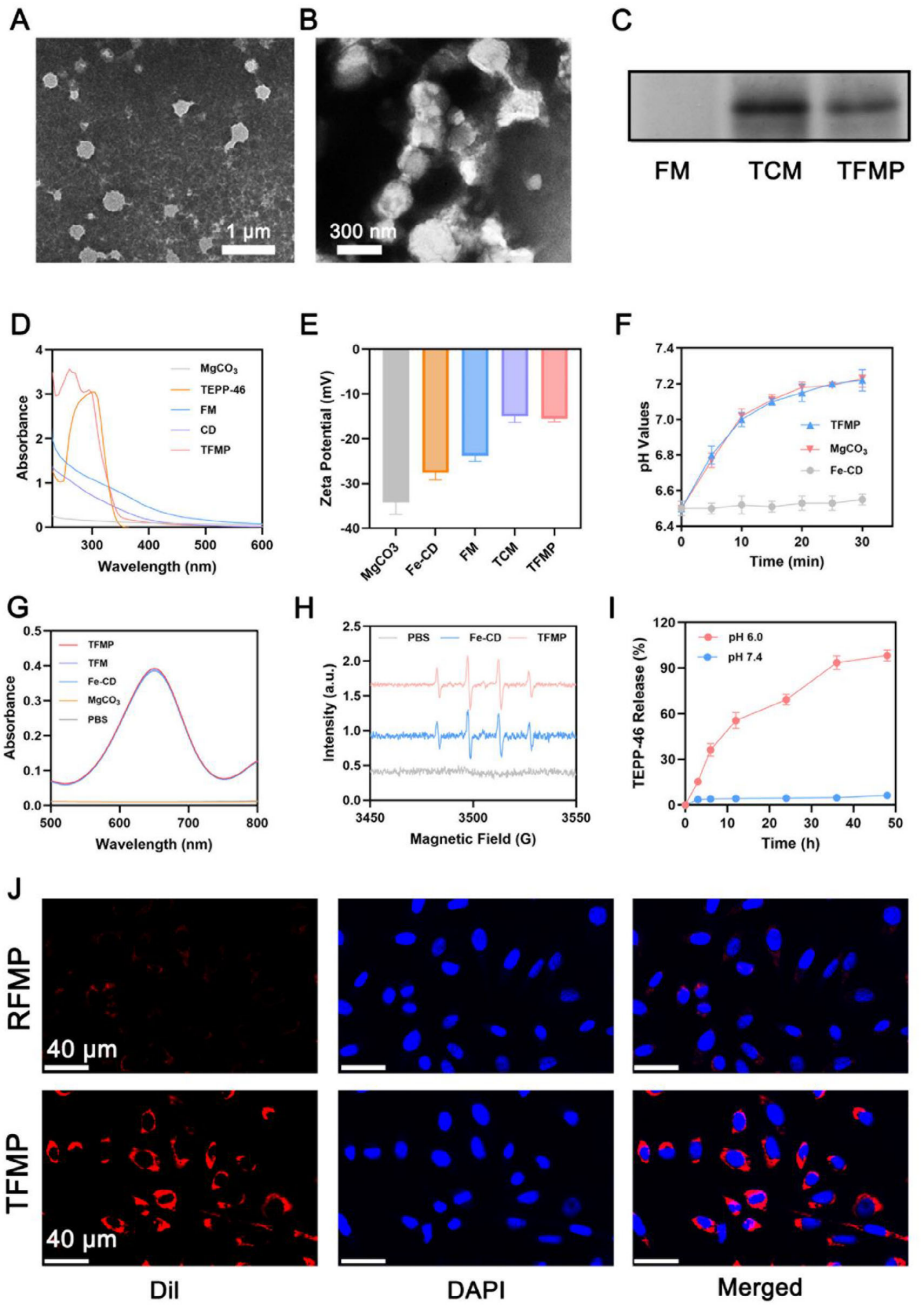
A) TEM image of T cell membranes (TCM) and B) TFMP. C) Western blot (WB) analysis of PD‐1 proteins in different formulations. D) Absorption spectra and E) Zeta potential of indicated formulations. Data are shown as the mean ± SD (*n* = 3). F) pH values of PBS after 0.5 h of incubation with different formulations. Data are shown as the mean ± SD (*n* = 3). G) UV–vis absorption spectra of oxTMB in different groups after co‐incubation with 5 µg mL^−1^ TMB and 10 mm H_2_O_2_. H) The generation of •OH by various formulations containing 10 mm H_2_O_2_ was assessed using Electron Paramagnetic Resonance (EPR). I) TEPP‐46 release profile under different pH value form TFMP. Data are shown as the mean ± SD (*n* = 3). J) CLSM images of cancer cells incubated with Dil labeled RFMP (Red blood cell membrane‐coated FM/TEPP‐46 nanoparticles) or TFMP for 1 h. Red: Dil, Blue.

According to previously published protocols, T‐cell membranes (TCM) were extracted.^[^ ^7^ ^]^ Subsequently, TCM co‐loaded with FM/TEPP‐46 to form TFMP. In brief, the TCM was mixed with FM and TEPP‐46, followed by ultrasonication using an ultrasound machine under ice‐cooled conditions. The resulting mixture was then extruded through a polycarbonate membrane for 10 cycles to ensure uniform vesicle formation. As a control, TCM‐coated FM (TFM) and TCM coated MgCO_3_ (TM) were generated. Transmission electron microscopy (TEM) revealed that the surface of the nanosheets was coated with a membrane‐like structure resembling TCM (**Figure** **2**A,B). Western blot analysis demonstrated that both TCM and TFMP exhibited high expression levels of PD‐1 protein (Figure 2C). UV–vis absorption spectroscopy showed characteristic peaks corresponding to TEPP‐46 and FM in TFMP, indicating effective drug encapsulation (Figure 2D). A similar surface potential of TCM and TFMP further confirmed successful TCM coating (Figure 2E). The particle size distribution is shown in Figure S3A (Supporting Information). The particle size of TFMP is slightly larger than that of FM, indicating the presence of surface cell membranes. TFMP shows good stability (Figure S3B, Supporting Information). As shown in Figure S4 (Supporting Information), the fluorescence co‐localization experiment and SDS‐PAGE experiment proved that the cell membrane was successfully encapsulated. Further investigation revealed that, in a mildly acidic environment (pH 6.5), the addition of MgCO_3_ and TFMP led to a pH increase to near neutrality (pH = 7.2), likely due to the reaction between MgCO_3_ and H⁺. This observation further supported that TFMP could react with H⁺ and the successful incorporation of MgCO_3_ into TFMP (Figure 2F). Given that peroxidase‐like enzymes can catalyze the generation of highly cytotoxic ·OH from H_2_O_2_ under acidic conditions, the peroxidase‐like (POD‐like) activity of TFMP was evaluated by monitoring the colorimetric reaction between ·OH and 3,3′,5,5′‐Tetramethylbenzidine (TMB). As expected, Fe‐CDs, TFM, and TFMP all catalyzed the oxidation of TMB to oxTMB, resulting in a visible color change at 652 nm. In contrast, no color change was observed when PBS or MgCO_3_ was incubated with H_2_O_2_ alone (Figure 2G; Figure S5, Supporting Information). Moreover, Electron Paramagnetic Resonance (EPR) spectroscopy clearly detected the characteristic four‐line signal (1:2:2:1) of ·OH in the presence of H_2_O_2_, confirming that Fe‐CDs and TFMP could effectively induce ·OH generation (Figure 2H). These findings collectively indicate that TFMP possesses robust peroxidase‐like activity. The pH‐dependent release behavior of TEPP‐46 from TFMP was further investigated. As shown in Figure 2I and Figure S6 (Supporting Information), the release rate of TEPP‐46 and Mg^2+^ significantly increased under acidic conditions, demonstrating that TFMP, with its acid‐responsive degradation properties. Under acidic conditions, TFMP obviously lost its original structure and underwent degradation, with a reduction in particle size (Figure S7, Supporting Information). It is well established that activated T cells highly express PD‐1 on their surface, which interacts with PD‐L1‐a receptor predominantly expressed on tumor cells‐to trigger T cell apoptosis and enable tumor immune evasion. Inspired by this mechanism, the TCM‐coated nanomaterials developed in this study are capable of targeting PD‐L1 on tumor cells, thereby enhancing the tumor‐specific accumulation of TFMP. Furthermore, the PD‐L1/PD‐1 interaction facilitates the internalization of TFMP into tumor cells, potentially reducing the immunosuppressive by downregulating PD‐L1 expression. To evaluate the tumor‐targeting capability of TFMP, we prepared red blood cell membrane‐encapsulated nanoparticles loaded with FM and TEPP‐46 (RFMP) as a control. Confocal laser scanning microscopy (CLSM) was employed to assess the targeting efficiency of different nanomaterials toward cancer cells. As shown in Figure 2J, TFMP exhibited significantly enhanced tumor cell targeting compared to RFMP, confirming that the PD‐1‐overexpressing TFMP nanomaterials can serve as promising candidates for targeted cancer therapy. The Co‐immunoprecipitation results showed that both TFMP and free PD‐L1 were detected in the input group (Figure S8, Supporting Information), indicating the presence of these two proteins in the samples. In the IP group, no bands were shown in the IgG group, ruling out the possibility of non‐specific binding. In the anti‐PD‐L1 group, bands were displayed at the positions of both PD‐1 and PD‐L1, suggesting that these two proteins were present in the precipitated complex. This indicates that TFMP interacts with PD‐L1 protein, which explains why TFMP exhibits better tumor targeting than RFMP.

### 2.3 In Vitro Anti‐Tumor Ability of TFMP

**Figure 3:**
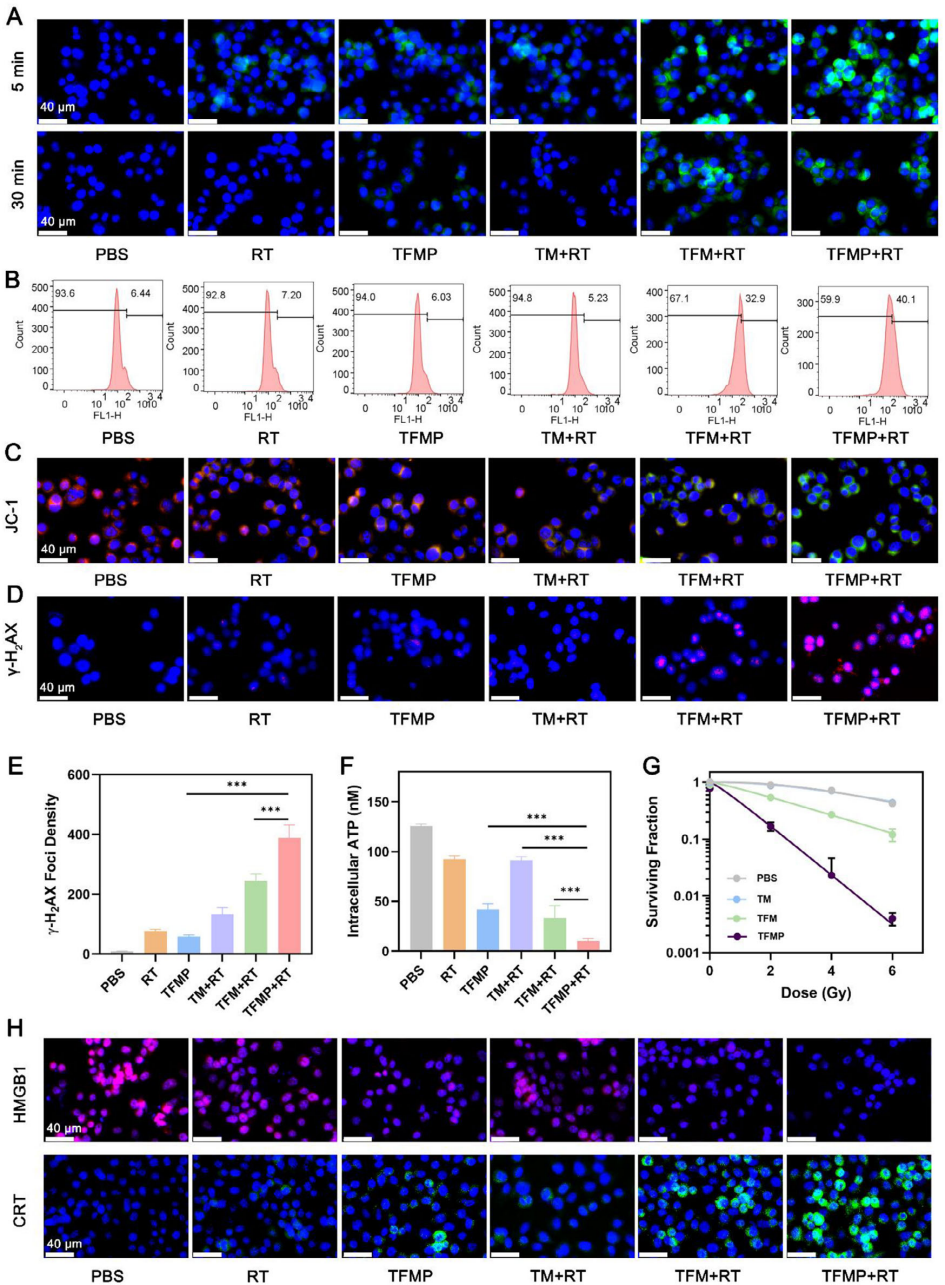
A) CLSM images and of •OH (green fluorescence) generated in 4T1 cells upon different times after treatments. RT: 4 Gy. B) •OH fluorescence intensity of Figure 2A (30 min). C) JC‐1 fluorescence analysis of 4T1 cells treated with different components(red: JC‐1 aggregates; green: JC‐1 monomer. RT: 4 Gy). D) Expression of γ‐H_2_AX (red fluorescence) and (E) γ‐H_2_AX Foci density from 4T1 cells following treatment with different formulations. RT: 4 Gy. Data are shown as the mean ± SD (n = 3). F) The intracellular ATP content after different treatments. RT: 4 Gy. Data are shown as the mean ± SD (*n* = 3). G) Clonogenic survival assay of 4T1 cells treated with different formulations under a series of radiation doses at 0, 2, 4, 6, and 8 Gy. MgCO_3_ concentration was 0.1 mg mL^−1^. Data are shown as the mean ± SD (*n* = 3). H) Expression of HMGB1 and CRT following treatment with different formulations. Statistical significance was calculated via one‐way ANOVA with Tukey's test: ^***^ *p* < 0.001.

Given the robust physicochemical properties, we investigated the anti‐tumor efficacy of TFMP in vitro. Previous studies have demonstrated that RT‐induced tumor hypoxia can upregulate NOX4 expression and promote H_2_O_2_ production,^[^ ^43^ ^]^ thereby creating a prerequisite for TFMP to generate abundant ROS within tumor cells. However, the high intracellular levels of glutathione (GSH) in tumor cells would scavenge ROS generated by RT, thereby attenuating the cytotoxic effects. In this nano‐system, TFMP loaded with Fe‐CD nanozymes can enhance ROS generation following RT through a chemical cascade reaction. The production of ·O_2_^−^ and H_2_O_2_ in tumor cells was shown in Figure S9 (Supporting Information). As shown in Figure S10 (Supporting Information), TFMP significantly increased intracellular pH. The production of ·OH in tumor cells after various treatments was assessed using CLSM. As shown in **Figure** **3**A, compared with the RT, TFMP, and TM+RT groups, the TFM+RT and TFMP+RT groups induced significantly higher levels of ·OH in tumor cells. Notably, even after 30 min of treatment, both TFM+RT and TFMP+RT groups exhibited strong ·OH and ROS fluorescence signals (Figure 3B; Figure S11, Supporting Information), indicating their superior catalytic activity in converting H_2_O_2_ into ·OH. This enhanced ROS generation may be attributed to the catalase‐like activity of Fe‐CD nanozymes, which enables a more sustained production of ROS. Mitochondrial membrane potential was evaluated using JC‐1 staining. The TFMP+RT group exhibited the lowest mitochondrial membrane potential and the most pronounced mitochondrial damage, as evidenced by the increased green fluorescence (Figure 3C). These results suggest that TFMP+RT primarily first damage mitochondria, leading to impaired ATP production and subsequent tumor cell damage. This hypothesis was further supported by measurements of intracellular ATP levels after different treatments (Figure 3F). Following TFMP+RT treatment, 4T1 cells exhibited extensive DNA damage, as confirmed by the marked induction of γ‐H2AX foci formation (Figure 3D,E). To further evaluate the radiosensitizing effects of different treatments, the survival fraction (SF) of 4T1 cells from different radiation doses was then analyzed. As shown in Figure 3G, TFMP most effectively enhanced the anti‐tumor efficacy of RT, and this radiosensitizing effect exhibited a dose‐dependent relationship with X‐ray exposure. Specifically, as the radiation dose increased, the cell survival fraction in the TFMP group significantly decreased. Live/dead cell staining, cytotoxicity assays, and flow cytometry‐based apoptosis analysis collectively demonstrated the cytotoxic effect of TFMP combined withRT on 4T1 cells (Figure S12, Supporting Information). TFMP also demonstrated low cytotoxicity toward mouse macrophages (Figure S13, Supporting Information), confirming its good biocompatibility. Moreover, effective ROS generation can trigger ICD, which activates the release of damage‐associated molecular patterns (DAMPs), such as calreticulin (CRT) and high mobility group box 1 (HMGB1). As expected, compared with other control groups, the TFMP+RT group showed a marked reduction in nuclear HMGB1 levels and increased surface exposure of CRT (Figure 3H; Figure S14, Supporting Information), providing strong evidence for the induction of ICD by TFMP+RT.

**Figure 4:**
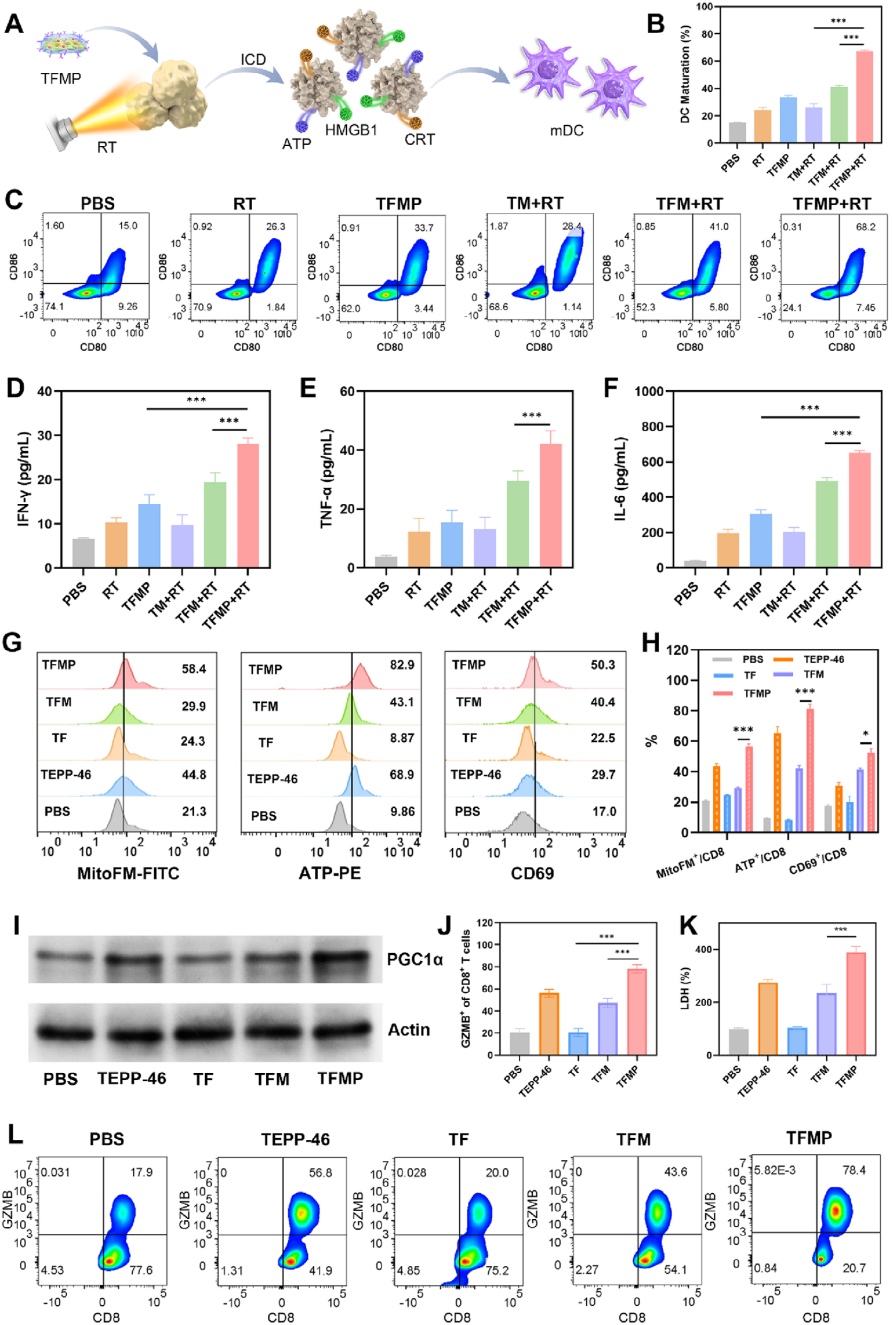
A) Schematic illustration of maturation of BMDCs in vitro. B) Quantitative analysis and C) Flow cytometry analysis of mature BMDCs after different treatments. Data are shown as the mean ± SD (*n* = 3). D) The levels of IFN‐γ, E) TNF‐α, and F) IL‐6 are secreted by mature BMDCs. Data are shown as the mean ± SD (*n* = 3). G) Fluorescence‐activated cell sorting (FACS) micrograph and H) statistical analysis of MitoFM, mitochondrial ATP, and CD69 expression in nanoparticles‐treated CD8^+^ T cells. TF: TCM+Fe‐CD. Data are shown as the mean ± SD (*n* = 3). I) Western blot analysis of PGC1a expression in nanoparticles‐treated CD8^+^ T cells at 48 h post activation. J) Quantification of granzyme B (GZMB) in CD8^+^ T cells after different treatments was analyzed by the flow cytometry. Data are shown as the mean ± SD (*n* = 3). K) Measurement of LDH levels in the supernatant after co‐incubation of the splenic T lymphocytes with 4T1 tumor cells for 24 h. Data are shown as the mean ± SD (*n* = 3). L) Representative plots of granzyme B (GZMB) in CD8^+^ T cells after different treatments. MgCO_3_ concentration was 0.1 mg mL^−1^. Statistical significance was calculated via one‐way ANOVA with Tukey's test: ^*^ *p* < 0.05. ^***^ *p* < 0.001.

The activation of antigen‐presenting cells (APCs) is a critical initiating step in adaptive anti‐tumor immunity.^[^ ^44^ ^]^ Among APCs, DCs are considered the most potent and play a central role in orchestrating adaptive immune responses.^[^ ^45^, ^46^ ^]^ During ICD, tumor cells release damage‐associated molecular patterns (DAMPs), which act as danger signals to activate DCs and enhance T cell‐mediated anti‐tumor immunity.^[^ ^47^ ^]^ We assessed the maturation of bone marrow‐derived dendritic cells (BMDCs) by measuring the expression levels of co‐stimulatory signals CD80 and CD86. As shown in **Figure** **4**A–C, the proportion of CD80^+^CD86^+^ BMDCs in the TFMP+RT group was 1.66‐folds, 2.40‐ folds, 2.02‐ folds, 2.59‐ folds, and 4.57‐folds higher than that in the TFM+RT, TM+RT, TFMP, RT, and PBS groups, respectively. Furthermore, TFMP+RT treatment induced the most pronounced secretion of pro‐inflammatory cytokines, including interferon‐γ (IFN‐γ), tumor necrosis factor‐α (TNF‐α), and interleukin‐6 (IL‐6) (Figure 4D–F). These findings confirm that TFMP+RT‐induced tumor cells effectively promote BMDCs maturation. To further investigate the immune‐activating mechanism of TFMP, CD8+ T cells isolated from spleens were stimulated with Ova peptide in the presence of five different treatments for 48 h: (1) PBS; (2) TEPP‐46; (3) TM; (4) TFM; and (5) TFMP. Flow cytometry was employed to evaluate the expression of MitoFM (a marker of mitochondrial activity), mitochondrial ATP (a key energy source for T cells), and CD69 (an early activation marker of T cells) in CD8+ T cells. As shown in Figure 4G,H, compared with the PBS and TF groups, TEPP‐46 and TFM treatments significantly increased MitoFM fluorescence intensity, ATP production, and CD69 expression, indicating that MgCO_3_‐based treatments (TEPP‐46 and TFM) can enhance T cell activation and mitochondrial function, thereby promoting ATP synthesis. Notably, the TFMP group exhibited the most pronounced effects, suggesting that TFMP induces the strongest mitochondrial metabolic reprogramming in CD8+ T cells. Peroxisome proliferator‐activated receptor γ coactivator‐1α (PGC1α) is a key regulator of mitochondrial function, which maintains mitochondrial structural and functional integrity by promoting mitochondrial biogenesis and autophagy to eliminate damaged mitochondria.^[^ ^48^, ^49^, ^50^ ^]^ Western blot (WB) analysis was performed to examine PGC1α expression in CD8+ T cells following different treatments. As shown in Figure 4I, PGC1α expression was significantly higher in the TFMP group compared to the control groups, indicating that TFMP best preserves mitochondrial functional integrity in CD8^+^ T cells. We further evaluated the impact of TFMP stimulation on T cell cytotoxicity against tumor cells. GZMB^+^CD8^+^ T cells are characterized by high expression of granzyme B, a key mediator of cytotoxic activity, and represent a highly activated subset capable of recognizing and eliminating tumor cells.^[^ ^51^, ^52^ ^]^ Analysis of GZMB expression revealed a significant increase in GMZB‐positive tumor‐infiltrating CD8^+^ T cells after TFMP treatment (Figure 4J,L). These T lymphocytes were co‐cultured with 4T1‐OVA tumor cells for 24 h, and the level of lactate dehydrogenase (LDH) released into the supernatant confirmed that TFMP‐treated T cells exhibited the strongest cytotoxic activity against 4T1‐OVA cells (Figure 4K). In addition, TFMP in combination with RT effectively inhibits OCR in 4T1 cells, while TFMP enhances OCR in CD8^+^ T cells (Figure S15, Supporting Information), indicating that it can suppress mitochondrial respiration in tumors and promote mitochondrial metabolism in T cells. These results demonstrate that TFMP treatment effectively activates antigen‐specific T cells to kill tumor cells.

### 2.4 In Vivo Anti‐Tumor Ability of TFMP

**Figure 5:**
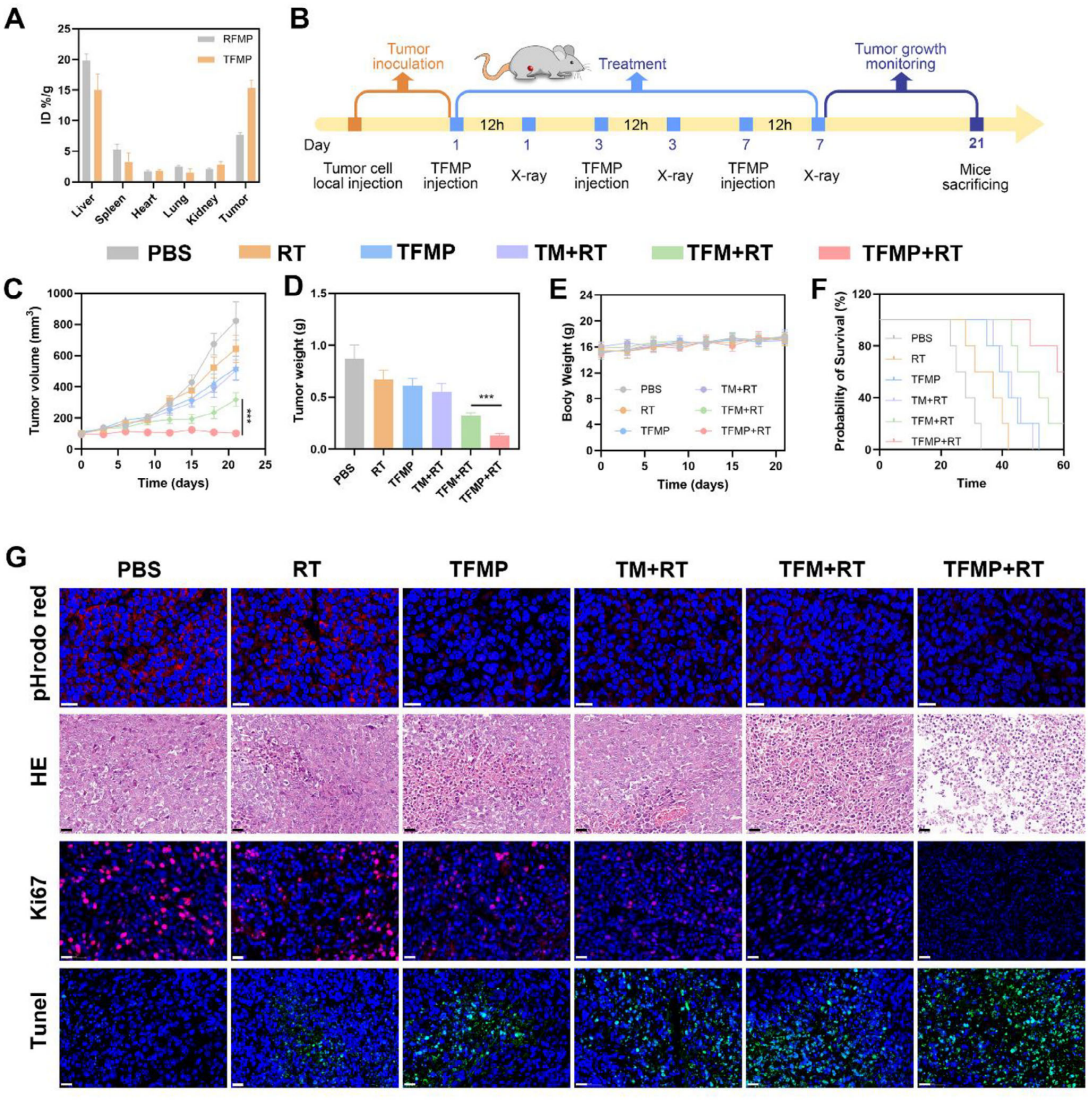
A) Biological distribution following the injection of RFMP or TFMP. Data are shown as the mean ± SD (*n* = 3). B) A schematic diagram outlining the timeline for tumor inoculation, treatment method, and monitoring of tumor growth. C) Tumor volumes were measured every 3 days across all groups. Data are shown as the mean ± SD (*n* = 5). D) Tumor weights were recorded for each treatment at the end of the study. Data are shown as the mean ± SD (*n* = 5). E) Body weight was measured every 3 days across all groups. Data are shown as the mean ± SD (*n* = 5). F) Survival curves after treatment. G) Fluorescence images of tumor tissues showing detection of pHrodo red, HE, TUNEL, and Ki‐67 staining after the indicated treatments. Scale bars: 40 µm. TEPP‐46 dose: 20 mg kg^−1^. Statistical significance was calculated via one‐way ANOVA with Tukey's test: ^***^ *p* < 0.001.

The in vitro therapeutic effect of TFMP combined with RT is promising, prompting further investigation into its in vivo application. Initially, the biodistribution of TFMP after intravenous injection was analyzed at various time points using inductively coupled plasma atomic emission spectroscopy (ICP‐AES). As shown in **Figure** **5**A, TFMP exhibited greater accumulation in the primary tumor at 12 h post‐injection. Therefore, RT was administered 12 h after TFMP injection. When tumor volume reached ≈ 100 mm^3^, tumor‐bearing mice were randomly assigned to six experimental groups (*n* = 5 per group): (1) PBS; (2) RT (4 Gy); (3) TFMP; (4) TM + RT; (5) TFM + RT; and (6) TFMP + RT. Treatments were administered on days 1, 3, and 7. Tumor volume and body weight were measured every 3 days, and survival rates were recorded (Figure 5B). During the 15‐day observation period, the PBS group exhibited rapid tumor growth, while both RT and TFMP showed moderate tumor suppression. However, monotherapy failed to completely inhibit tumor progression. Combination therapies, such as TM + RT and TFM + RT, further improved survival rates. Notably, the TFMP + RT group demonstrated the most significant tumor growth inhibition, with an 80% survival rate sustained over 60 days (Figure 5C,D,F), confirming the potent in vivo antitumor efficacy. Throughout the treatment period, no significant body weight loss was observed (Figure 5E), and no pathological changes or biochemical abnormalities were detected in major organs (Figures S16 and S17, Supporting Information), demonstrating the excellent biocompatibility and safety profile of the treatment. Previous studies have shown that nanomedicines are easily captured by the mononuclear‐phagocyte system (such as the liver, spleen, and lungs), leading to non‐targeted accumulation.^[^ ^53^ ^]^ Surface charge (such as negative charge) and lipophilicity affect the adsorption of opsonins, thereby altering the clearance rate.^[^ ^54^, ^55^, ^56^, ^57^, ^58^ ^]^ Additionally, long‐term use of nanomedicines may lead to immunosuppression or autoimmune‐like reactions, such as the delayed organ toxicity observed in ICIs treatment.^[^ ^59^, ^60^ ^]^ TFMP, due to its cell membrane biomimetic characteristics, has less accumulation in major organs and is a relatively safe material. However, its potential immunotoxicity needs to be further evaluated in the future. Subsequently, the pH‐sensitive fluorescent probe phrodo red, which exhibits enhanced fluorescence in acidic environments, was employed to assess tumor microenvironmental acidity. Compared to the PBS and RT groups, mice treated with TM + RT, TFM + RT, and particularly TFMP + RT showed significantly reduced fluorescence signals in tumor tissues under physiological conditions (Figure 5G). This suggests that the carbonate components in TM, TFM, and TFMP effectively neutralize hydrogen ions, thereby alleviating tumor acidity. The hematoxylin and eosin (HE), Ki‐67 proliferation staining, and Terminal deoxynucleotidyl transferase‐mediated dUTP nick end labeling (TUNEL) confirmed that TFMP + RT induced extensive tumor necrosis and apoptosis.

**Figure 6:**
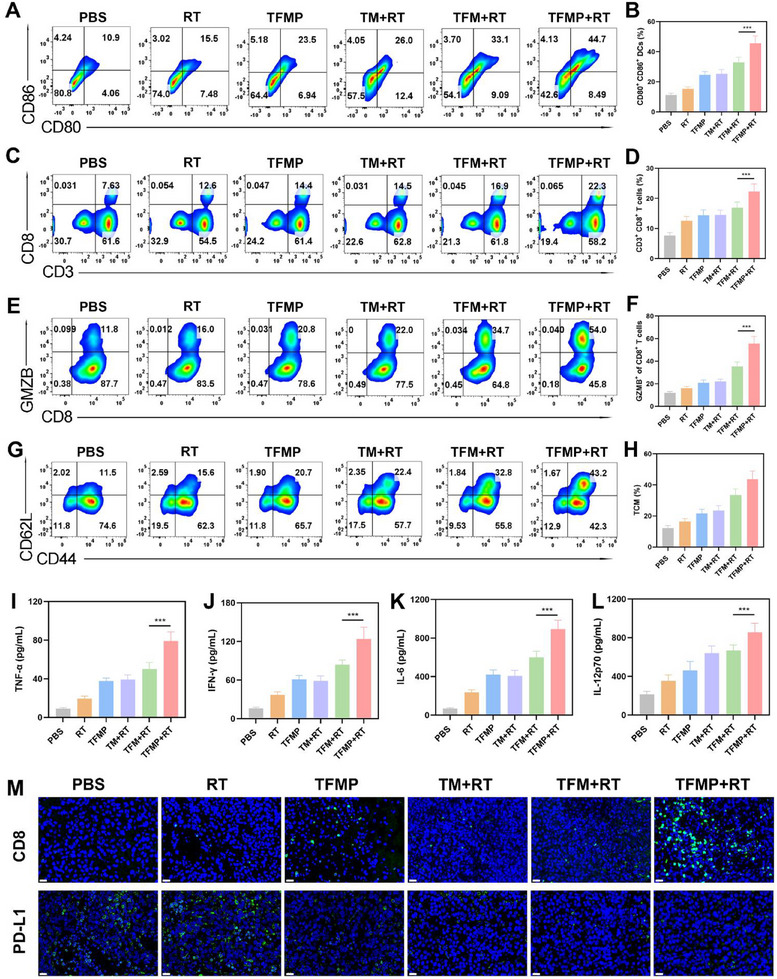
A) Flow cytometry and B) Quantitative analysis showing the effects of treatments on DCs maturation in lymph nodes. Data are shown as the mean ± SD (*n* = 5). C) Flow cytometry and D) Quantitative analysis showing infiltration of CD3^+^ CD8^+^ T lymphocytes in 4T1 tumor tissues. Data are shown as the mean ± SD (*n* = 5). E) Flow cytometry and F) Quantitative analysis showing GZMB^+^ cells among CD8^+^ T cells in tumor tissues. Data are shown as the mean ± SD (*n* = 5). G) Flow cytometry and H) Quantitative analysis showing CD44^+^CD62L^+^ cells among CD8^+^ T cells in blood. Data are shown as the mean ± SD (*n* = 5). I) Secretion of pro‐inflammatory cytokines, including TNF‐α, J) IFN‐γ, K) IL‐6, and L) IL‐12p70 in sera after exposure to different treatments. Data are shown as the mean ± SD (*n* = 5). M) CD8 and PD‐L1 staining of tumor sections after the indicated treatments. Scale bars: 40 µm. Statistical significance was calculated via one‐way ANOVA with Tukey's test: ^***^ *p* < 0.001.

To investigate the impact of combined therapy on anti‐tumor immunity, we collected lymph nodes and spleens from mice after various treatments to assess DCs maturation and T cell activation. As shown in **Figure** **6**A,B, compared with the PBS group (10.9%), the expression of co‐stimulatory markers CD80 and CD86 on DCs in the RT group showed a slight increase (15.5%), while the TFMP and TM+RT groups exhibited significantly higher expression levels, reaching 23.5% and 26.0%, respectively. The TFM+RT group showed an even greater increase, with CD80^+^ CD86^+^ expression reaching 33.1%. Notably, the TFMP+RT group demonstrated the most pronounced increase, with CD80^+^ CD86^+^ expression reaching 44.7%, markedly higher than in all other control groups. Next, we evaluated the proportion of CD3^+^CD8^+^ T cells. The TFMP+RT group showed the highest proportion of CD8^+^ T cells (22.3%), which was 2.92‐folds higher than that in the PBS group (7.63%), 1.77‐folds higher than in the RT group (12.6%), 1.59‐folds higher than in the TFMP group (14.4%), 1.54‐folds higher than in the TM+RT group (14.5%), and 1.32‐folds higher than in the TFM+RT group (16.9%) (Figure 6C,D). Furthermore, flow cytometry analysis revealed that the number of CD8^+^ GMZB^+^ T cells in the TFMP+RT group increased by more than 4.5‐fold compared to the PBS group (Figure 6E,F). Central memory T cells (TCM) are an important subset of memory T cells, serving as a “reservoir” to provide support for subsequent immune responses and long‐lasting anti‐tumor immunity.^[^ ^61^, ^62^ ^]^ We evaluated the proportion of TCM in the blood of mice in each group after treatment, as shown in Figure 6G,H. The TFMP+RT group showed the highest TCM ratio, demonstrating its long‐term anti‐tumor ability. Additionally, following combination therapy, serum levels of pro‐inflammatory cytokines, including TNF‐α, IFN‐γ, IL‐6, and IL‐12p70, were significantly elevated, indicating that TFMP induced the most robust anti‐tumor immune response (Figure 6I–L). To further validate these findings, immunofluorescence staining was performed on tumor tissues. As shown in Figure 6M, CD8^+^ T cell infiltration and distribution were markedly increased in tumor tissues from mice treated with TFMP combined with RT, which may account for the effective tumor growth inhibition observed in this group. Notably, RT alone induced PD‐L1 upregulation, it also facilitated greater accumulation of TFMP in tumor tissues, where it bound to PD‐L1 on tumor cell surfaces. Therefore, the combination of TFMP and RT resulted in PD‐L1 downregulation. These results strongly suggest that the upregulation of PD‐L1 expression following RT creates an ideal target for TFMP to specifically accumulate in tumor tissues. Moreover, the binding of TFMP to tumor cells can block PD‐L1 expression on their surface, thereby removing a major barrier to T cell‐mediated tumor cell killing. This mechanism contributes to enhancing the overall potential of anti‐tumor immunity.

**Figure 7:**
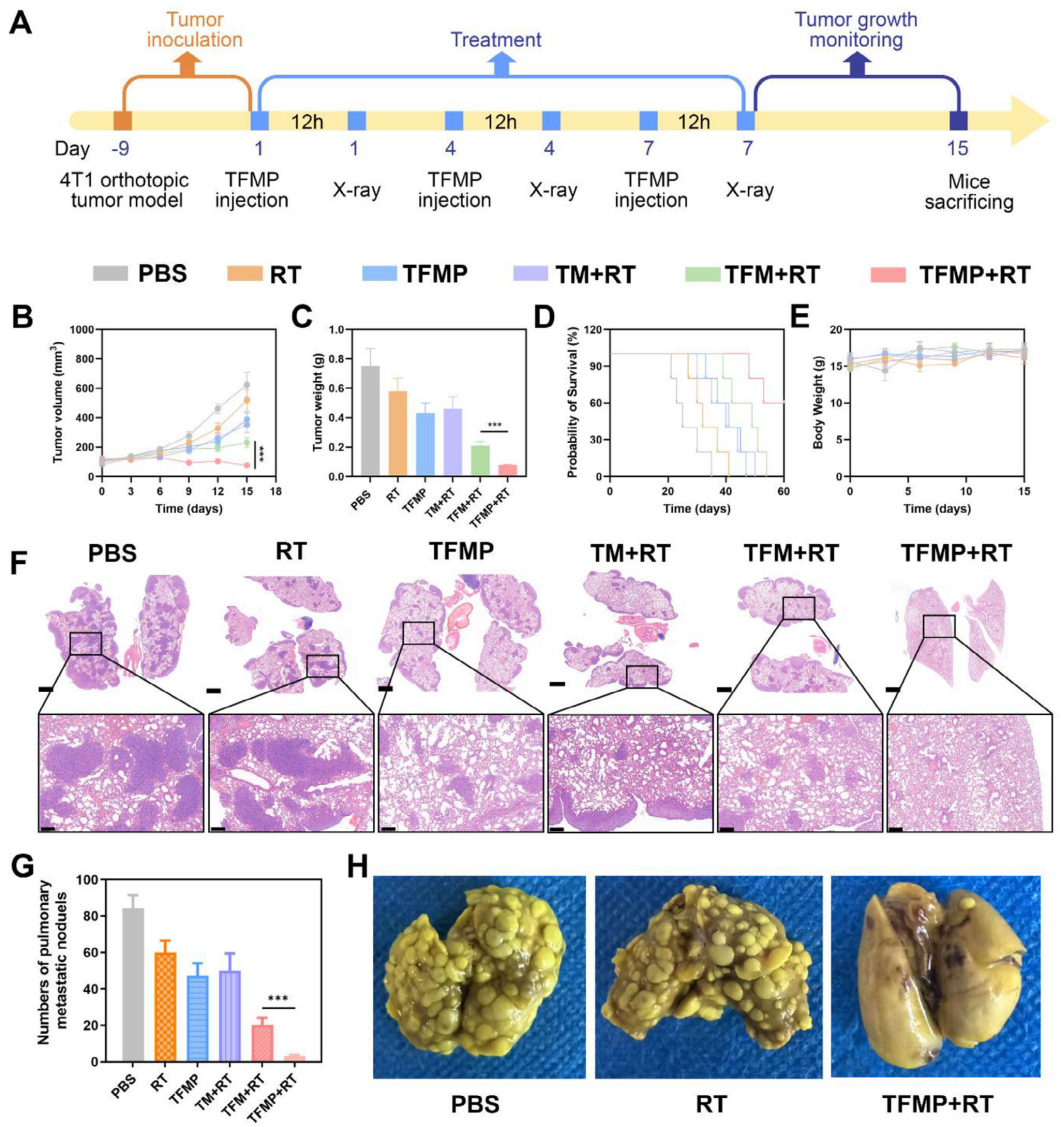
A) Schematic illustration of the studies of 4T1 tumor metastasis. B) Evolution of the tumor volume bearing mice after various treatments. Data are shown as the mean ± SD (*n* = 5). C) Tumor weight after treatment in different groups. Data are shown as the mean ± SD (*n* = 5). D) Survival curves after treatment. E) body weight change of the tumor volume bearing mice after various treatments. Data are shown as the mean ± SD (*n* = 5). F) HE staining of the lung tissues. Scale bars: 1000 µm (upper) and 200 µm (below). G) Quantitative analysis of pulmonary metastatic nodules for every group. Data are shown as the mean ± SD (*n* = 5). H) The images of the collected lungs. TEPP‐46 dose: 20 mg kg^−1^. Data are shown as the mean ± SD (*n* = 5). Statistical significance was calculated via one‐way ANOVA with Tukey's test: ^***^ *p* < 0.001.

Distant organ metastasis remains a leading cause of mortality in breast cancer patients. Given the strong efficacy of TFMP combined with RT in suppressing tumor progression and activating anti‐tumor immune responses, we further evaluated its long‐term effect on metastasis inhibition in a murine model. As illustrated in **Figure** **7**A, a subcutaneous 4T1 breast tumor model was established. When the tumor volume reached ≈ 100 mm^3^, tumor‐bearing mice were randomly assigned to six experimental groups (n = 5 per group) and received the following treatments: (1) PBS; (2) RT (4 Gy); (3) TFMP; (4) TM + RT; (5) TFM + RT; and (6) TFMP + RT. Treatments were administered on days 1, 4, and 7. Tumor volume and body weight were monitored every 3 days, and survival rates were recorded. During the 15‐day observation period, the TFMP + RT exhibited significantly reduced tumor volume and weight compared to the control groups, demonstrating its potent therapeutic effect on primary breast cancer. Subsequently, lung tissues were collected for macroscopic imaging and HE staining. As shown in Figures 7F,H, both the PBS and RT groups displayed evident metastatic lesions in the lungs. In contrast, the TFMP + RT group showed a near‐complete absence of metastatic nodules. These results indicate that TFMP combined with RT effectively prevents the long‐term metastasis of residual breast cancer cells to the lungs.

## 3 Conclusion

In summary, we have developed a biomimetic nanosystem co‐loaded with MgCO_3_/Fe‐CD and TEPP‐46 onto T lymphocyte membranes, designed to simultaneously induce mitochondrial metabolic reprogramming in both T cells and tumor cells. Following intravenous administration, TFMP specifically targets tumor tissues with high PD‐L1 expression, competitively binds to PD‐L1, and thereby alleviates immune checkpoint‐mediated immunosuppression of T cells. Under external X‐ray irradiation, TFMP continuously catalyzes the conversion of RT‐generated H_2_O_2_ into hydroxyl radicals •OH, enabling sustained ROS production. Consequently, the combination of TFMP and RT effectively induces mitochondrial damage and immunogenic cell death in tumor cells, leading to the release of DAMPs, which promote DCs maturation and T cell infiltration. Furthermore, the acid‐neutralizing reaction between magnesium carbonate in TFMP and tumor‐derived hydrogen ions mitigates the acidic tumor microenvironment. The released magnesium ions and TEPP‐46 further enhance T cell activation and mitochondrial function, promoting ATP and GMZB production, thereby effectively eliminating residual tumor cells. TFMP not only modulates the tumor microenvironment and generates ROS to sensitize RT but also functions as a tumor immunomodulator that enhances radioimmunotherapy, demonstrating significant potential for clinical translation. In future studies, we will focus on further optimizing TFMP's ability to regulate the immunosuppressive tumor microenvironment, particularly its capacity to alleviate post‐RT immunosuppression and achieve long‐term antitumor effects when combined with RT.

## Conflict of Interest

The authors declare no conflict of interest.

## Supporting information



Supporting Information

## Data Availability

The data that support the findings of this study are available from the corresponding author upon reasonable request.
