# Supplementary material for: Differentiated T Lymphocytes and Cancer Cell Mitochondrial Metabolism to Enhance Radioimmunotherapy by a Biomimetic Nanozyme System
Source: Adv Sci (Weinh). 2025 Nov 3;13(3):e15097. doi: 10.1002/advs.202515097 (PMC12806320; doi:10.1002/advs.202515097)
Supplement: Supplementary file 1 — Supporting Information [file ADVS-13-e15097-s001.docx]

Supporting Information

**Differentiated T lymphocytes and cancer cell mitochondrial metabolism to enhance** **radioimmunotherapy by a biomimetic nanozyme system**

*Hanyu Zhang,* *Yuhan Deng, Yantong Lu, Miao Wang, Kun Qiao, Zifan Yang, Shipeng Ning*, Tong Liu**

Dr. H. Zhang, Dr. Y. Deng, Dr. M. Wang, Dr. K. Qiao, and Prof. T. Liu

Department of Oncology Surgery, Harbin Medical University Cancer Hospital, Harbin, 150000, China.

E-mail: [liutong@hrbmu.edu.cn](mailto:liutong@hrbmu.edu.cn)

Dr. Y. Lu

Digestive Diseases Center, The Seventh Affiliated Hospital, Sun Yat-sen University, Shenzhen, 518107, China.

Dr. Z. Yang and Prof. S. Ning

Department of Breast Surgery, The Second Affiliated Hospital of Guangxi Medical University, Nanning, 530000 China.

Email: [nspdoctor@sr.gxmu.edu.cn](mailto:nspdoctor@sr.gxmu.edu.cn)

Prof. S. Ning

Research Center of Nanomedicine Technology, The Second Affiliated Hospital of Guangxi Medical University, Nanning, 530000, China.

Prof. T. Liu

NHC Key Laboratory of Cell Transplantation, Harbin Medical University, Harbin,150081, China.

**Experimental Procedures**

**Materials**

DCFH-DA, Dil, FITC-NH_2_**,** TEPP-46, and ELISA kit used in this work were purchased from Guangzhou Ruiao Biotechnology Co., Ltd.(China). Magnesium carbonate (MgCO_3_) nanosheets were purchased from Yumu (Ningbo) New Materials Co., LTD. All of the aqueous solutions were prepared using purified deionized (DI) water purified with a purification system (Direct-Q3, Millipore, USA). The other solvents used in this work were purchased from Sinopharm Chemical Reagent (China) and Shanghai Macklin Biochemical Technology Co., Ltd. (China).

**Preparation and characterization of Fe doped carbon dots (Fe-CD) loaded MgCO_3_ (FM), T cell membrane (TCM)-coated FM (TFM), TCM coated MgCO_3_ (TM) and TCM-coated FM/TEPP-46 (TFMP)**

Fe-CD were synthesized by a one-step hydrothermal method according to the literature^[1]^. Based on the previous method, the TCM were obtained from EL4 cells^[2]^. Then, aqueous solution of MgCO_3_ (10 mg/mL, 1mL) and Fe-CD (2 mg/mL, 1mL) was mixed and sonicated (12 W output) for 3 min. Then the FM precipitation was obtained by centrifugation (8000 rpm, 5 min). Then, 2 mg of FM and 1 mg TEPP-46 were mixed with 1mg TCM and then repeatedly coextruded through 400 nm pores. The resultant TFMP particles were centrifuged and washed with PBS several times to remove the excess TM and TEPP-46. Red blood cell membrane-coated FM/TEPP-46 (RFMP) was prepared in the same way as TFMP. TFM was prepared in the same way as TFMP, except that TEPP-46 was not added. TM was prepared in the same way as TFMP, except that TEPP-46 and Fe-CD were not added. To label FM with FITC, FM was stirred with NH2-FITC in the dark for 2 hours. Then the FITC labeled FM was obtained by centrifugation (8000 rpm, 5 min).

TEPP-46 loading capacity were calculated by HPLC at the Agilent HPLC1260 II. Loading capacity = M_drug_/M_TFMP_. Where M refers to the mass. Protein expression was determined by western blot and Sodium Dodecyl Sulfate Polyacrylamide Gel Electrophoresis (SDS-PAGE). The particle size and zeta potential were measured by DLS. The morphology of synthesized materials was observed with field-emission TEM (JEM-F200). The thickness of nanomaterials was conducted using an Atomic Force Microscope (Dimension Edge, Bruker). XRD was measured on a D8 Advance (Bruker-AXS, Germany). XPS were measured using an ESCALAB 250Xi (Thermo Scientific, USA). FTIR was measured using Bruker Vertex 70.

**Cell culture**

4T1 mouse breast cancer cell line (RRID: CVCL_0125) was obtained from the Cell Bank of the Chinese Academy of Sciences and incubated in RPMI-1640 medium supplemented with 10% FBS in a humidified atmosphere at 37℃. RAW264.7 cells were obtained from Wuhan Procell Life Science & Technology Co., Ltd. The cells were cultured in DMEM medium supplemented with 10% fetal bovine serum (FBS) and 1% penicillin-streptomycin at 37 °C in a humidified incubator with 95% O_2_ and 5% CO_2_.

**Animal tumor models**

Female BALB/c mice aged 5-6 week were purchased from Vital River Company (Beijing, China). BALB/c mice were subcutaneously injected with 5 × 10^6^ 4T1 cells into the right flank (primary tumors). All animal procedures were performed in accordance with the guidelines for Care and Use of Laboratory Animals of the Ministry of Health in People’s Republic of PR China and approved by the Animal Ethics Committee of Guangxi Medical University (Approval number: 2025-KYL (012)).

**Detection of ROS**

The TMB Colorimetric Reaction experiment was used to detect the generation of hydroxyl radicals in TFMP. Typically, the TFMP (final TEPP-46 concentration was 5 μg/mL) was mixed with TMB (final concentration 5 μg/mL) in PBS buffer solution (pH 7.4). Subsequently, 10 mM H_2_O_2_ was added to the system. The absorbance of TMB was measured every 1 minute using a UV-vis spectrophotometer. Electron Paramagnetic Resonance (EPR) experiments were conducted on Bruker EMXplus EPR spectrometer.

**TEPP-46 release study**

The *in vitro* TEPP-46 release profile from TFMP was carried out. 10 mL of TFMP containing 20 μg TEPP-46 was added into culture dish. To investigate the stimuli effect of H^+^ on the release behavior, the release experiment of TEPP-46 was initially performed in PBS solution with a pH of 7.4 or 6.0. At appropriate time point, 100 μL of different samples were collected, and HPLC was used to monitor the released TEPP-46 content.

***In vitro* cancer targeting study**

4T1 cells were seeded in 24-well plates and cultured for 12 h. Then, 100 μL Dil labeled RFMP or TFMP (10 μg/mL TEPP-46) was added to the medium. Then the cells were incubated for 2 h at 37 °C, 5% CO_2_, and then washed with PBS three times. The cells were then fixed with PFA for 30 min at room temperature, stained with DAPI and then imaged by using a confocal laser scanning microscope (CLSM; IX81, Olympus, Japan).

For Co-immunoprecipitation, TFMP were mixed with free PD-L1 protein (MedChemExpress). The subsequent combined protein complex were subjected to immunoprecipitation with anti-PD-L1 antibody (Abcam) or normal IgG (Abcam) and protein A-Sepharose. Immune complexes were washed and subjected to immunoblotting with anti-PD-L1 (Abcam) or anti-PD-1 (Abcam) antibodies.

**Intracellular ·OH, ROS, Live-Dead Cell Staining,** **oxygen consumption rates (OCR), ATP content, mitochondrial membrane potential,** **apoptosis, immunogenic cell death (ICD) and cell cytotoxicity detection**

4T1 cells (1.5 × 10^5^ per well) were seeded in a 12-well plate for 12 h. Then the cells were incubated and treated with 6 different groups for 12 h: (1) PBS; (2) RT (4 Gy); (3) TFMP; (4) TM+RT; (5) TFM+RT and (6) TFMP+RT. MgCO_3_ concentration was 0.1mg/mL. After an additional 12 h, HPF, DCFH-DA and Mitochondrial Membrane Potential Assay Kit (with JC-1) were used according to the instruction. The intracellular ATP content was measured by employing a commercial assay kit (Beyotime) according to the instruction. Live-Dead Cell Staining was conducted by using Calcein-AM/PI Live/Dead Cell Double Staining Kit (Beijing Solarbio Science & Technology Co., Ltd) according to the instruction. Cell cytotoxicity was determined by the CCK-8 Cell Proliferation and Cytotoxicity Assay Kit (Beijing Solarbio Science & Technology Co., Ltd) according to the instruction.

The OCR of cells was measured by using a Seahorse instrument (XF24, Agilent) according to the literature^[3]^. Three baseline recordings were made, followed by sequential injection of 1 µM oligomycin, FCCP (0.25 µM, carbonyl cyanide-p-trifluoromethoxyphenylhydrazone) that uncoupled oxygen consumption from ATP production to obtain maximal OCR, and 0.5 µM rotenone/antimycin A that inhibited complex I and III.

Cellular apoptosis was assessed by plating 4T1 cells in 6-well plates and treating them via the six methods listed above for 12 h. After an additional 12 h, cells were harvested with EDTA-free trypsin, and annexin V-FITC/PI (Beyotime) was used to analyze cellular apoptosis via flow cytometry.

For ICD detection, the cells were washed with PBS three times, fixed with 4% PFA and permeabilized with 0.1% Triton X-100 for 10 min. After washing with PBS three times, the cells were blocked with 10% FBS, and incubated with Anti-Calreticulin antibody or Anti-HMGB1 antibody (Bioss) and fluorescent labeled secondary antibody for 30 min. The cells were washed with PBS three times, then stained with DAPI for 20 min. Finally, the cells were washed with PBS three times and observed using CLSM. For quantification of released HMGB1 in medium, the medium was collected after the cells were treated with materials. Then cell medium (20 μL) was used for ELISA detection.

**Intracellular pH detection**

4T1 cells (1.5 × 10^5^ per well) were seeded in a 12-well plate for 12 h. Then the cells were incubated and treated with 3 different groups: (1) PBS; (2) FM and (3) TFMP. MgCO_3_ concentration was 0.1mg/mL. Then, pHrodo Red (Invitrogen) were used for acidity detection according to the instruction.

**Intracellular H_2_O_2_ and ‧O₂⁻ Detection**

4T1 cells were seeded into a 6-well plate and cultured overnight. 4T1 cells were pre-incubated incubated and treated with 2 different groups: (1) PBS; (2) RT (4 Gy). The MgCO_3_ concentration was 0.1mg/mL. Reactive Oxygen Species Assay Kit for Superoxide Anion with DHE (Beyotime) and Hydrogen Peroxide Assay Kit (Beyotime) were used according to the instructions.

**DNA double-strand breaks (γ-H_2_AX immunofluorescence analysis) *in vitro***

4T1 cells were seeded in confocal dishes at a density of 1×10^5^ cells per dish for 24 h. Next, cells in each confocal dish were incubated with six different groups as mentioned above. The RT was conducted 2h after different treatment. Then the cells were fixed by 4% paraformaldehyde for 30 min, rinsed three times with PBS, treated with triton-X 100 for 10 min at room temperature to enhance cell permeabilization, and then rinsed three times with PBS again. The cells were exposed to a blocking buffer (1% BSA in PBS) for 1 h at room temperature and incubated with γ-H_2_AX antibody (dilution 1:500) overnight at 4 ºC. The next day, the cells were washed three times with PBS to remove the excess antibody and incubated with goat anti-mouse IgG (dilution 1:1000) for 1h at room temperature. Cells nuclei were stained by DAPI at room temperature. At last, the cells were imaged by confocal microscopy (Leica, Wetzlar, Germany).

**Cell toxicity**

Typically, 4T1 cells were incubated in plates at 37 °C with 5% CO_2_ for 24 h; afterward, the culture medium was replaced by new culture medium, cells were incubated with 6 different groups as mentioned above. After incubation for another 24 h. Finally, the viability of 4T1 cells was determined by a CCK-8 cell cytotoxicity assay kit (Beyotime) according to the instructions. The cell viability of RAW 264.7 cells after TFMP treatments with different MgCO_3_ concentrations was conducted using the same method.

**Clonogenic survival assay**

The effect of TFMP on the radiosensitivity of 4T1 cells was assessed by a clonogenic assay. 500 cells per flask were seeded in 25 cm^2^ flasks and cultured in normoxia for 24 h. Flasks were treated under following conditions: 1) PBS; 2) TM; 3) TFM and (4) TFMP. MgCO_3_ concentration was 0.1mg/mL. The cells were washed with PBS, and then exposed to 0, 2, 4 or 6 Gy of radiation in sealed flasks containing 5 mL of complete medium. To allow formation of colonies, after radiation, the cells were then incubated for another 10 days, without changing the media. To determine the clonogenic survival rate, cultures were first fixed with paraformaldehyde and then stained with trypan blue. Colonies with greater than 50 cells were counted under the microscope, and the survival fractions (SF) were calculated using the formula SF = colonies counted/cells seeded.

**Transwell experiment on Bone marrow-derived dendritic cells (BMDCs) stimulation in vitro**

BMDCs were isolated from 8-week-old BALB/c mice bone marrow. For BMDCs maturation assay, 1 × 10^5^ 4T1 cells were treated by the above four groups and then cocultured with 1 × 10^6^ BMDCs in the transwell culture system, and BMDCs were then isolated by anti-CD11c magnetic beads (Thermo Fisher). Then BMDCs were stained with FITC-anti-CD80 and PE-anti-CD86 (Abcam). Finally, the cells were sorted using flow cytometer (Beckman-Coulter, USA). The secretion levels of cytokines including TNF-α, IL-6, and IFN-γ in the samples were tested with ELISA kits.

**Cell isolation from spleens**

Spleens were aseptically isolated from mice and incubated at 37℃ in 10% FBS RPMI media containing 1.4 mg/ml collagenase A (Roche) and 30 μg/ml DNase I for 60 min. The treated lung tissue and spleen was dissociated over the 70 μm cell strainer (Fisherbrand). Strainer was washed to collect single-cell suspension. Red blood cells were lysed with ACK lysing buffer (Lonza) for 5 min following by washing of cells with culture media. Cells were counted and adjusted to 5 × 10^6^ cells/ml. Spleen cells were washed twice in fresh magnetic-activated cell sorting (MACS) buffer. CD8^+^ T cells were magnetically purified via negative selection using MACS cell separation system according to the manufacturer’s protocols.

**Western blot and T cells response measurements experiments**

CD8^+^ T cells from spleens were activated by Ova peptide (0.5 μM) in the presence of 5 groups for 48h: (1) PBS; (2) TEPP-46; (3) TM; (4) TFM and (5) TFMP. Each group of materials was pre-soaked in PBS solution with a pH of 6.0. MgCO_3_ concentration was 0.1mg/mL, followed by resting the cells in T cell medium with IL-2 (30 IU/ml) for 2 days, and then re-stimulation with IL-2+Ova peptide (0.5 μM) for 2 days. The OCR of cells was measured by using a Seahorse instrument (XF24, Agilent) according to the literature^[4]^. The expression of PGC1α and β-Actin was analyzed by western blot. The secretion of IL-2 in the supernatant and expression of MitoFM, mitochondrial ATP, CD69, IFN-γ and Granzyme B (GZMB) in CD8^+^ T cells was detected by ELISA and flow cytometry.The upper layer of the medium was added to the plates pre-seeded with 4T1-OVA tumor cells (3×10^4^ per well in 24-well plates), and the concentration of LDH in the supernatant were detected by LDH Assay Kit.

***In vivo* biodistribution study**

Female BALB/c mice aged 5-6 week were purchased from Vital River Company (Beijing, China). BALB/c mice were subcutaneously injected with 5 × 10^6^ 4T1 cells into the right flank. When tumors reached 300 mm^3^, tumor bearing mice (n = 3) received an intravenous (*i.v.*) injection of 100 μL PBS containing RFMP or TFMP (with a TEPP-46 dose of 20 mg/kg). Then the mice were sacrificed at different time after injection to collect the tumors and major organs. The content of magnesium ions was detected by ICP-AES.

***In vivo* anti-tumor study**

Female BALB/c aged 5-6 week were purchased from Vital River Company (Beijing, China). BALB/c mice were subcutaneously injected with 5 × 10^6^ 4T1 cells into the right flank. When the tumor grows to approximately 100 mm^3^, treatment is carried out on day 0. The mice were firstly divided randomly into different groups (Each group included 5 mice): (1) PBS; (2) RT (4 Gy); (3) TFMP; (4) TM+RT; (5) TFM+RT and (6) TFMP+RT. The MgCO_3_ dose was 40 mg/kg. Mice body weight and tumor volume in all groups were monitored every 3 days. A caliper was employed to measure the tumor length and tumor width, and the tumor volume was calculated according to following formula. Tumor volume = tumor length × tumor width^2^ / 2. After 21 days of treatment, mice were sacrificed. Five main organs (heart, liver, spleen, lung, and kidney) of all mice were harvested, washed with PBS, and fixed with paraformaldehyde for histology analysis. And the tumor tissues were weighed, and fixed in 4% neutral buffered formalin, processed routinely into paraffin, and sectioned at 4 μm. The primary tumor sections were stained with HE, TUNEL, Ki-67 and pHrodo red and finally examined by using fluorescence microscope (IX81, Olympus, Japan).

To examine DC maturation in vivo, the inguinal lymph nodes (LN) were harvested. The frequency of DC maturation in the LNs was then examined by CD11c^+^ cell sorting kit (Thermo Fisher) and flow cytometry after immunofluorescence staining with FITC-anti-CD80 and APC-anti-CD86 (Biolegend)**.** To study the T cells content and function in tumors, tumors were harvested from mice in different groups and treated with flow cytometry after immunofluorescence staining with FITC-anti-CD3 and PE-anti-CD8 (Abcam); FITC-anti-CD8 and APC-anti-GZMB (Biolegend). The blood samples were collected on day 14 after the first treatment for immune memory study. The CD8^+^ T lymphocytes were isolated by using the Mouse CD8^+^ T Cell Isolation Kit (Thermo Fisher), stained with antibodies (anti-CD62L-APC and anti-CD44-FITC). The subpopulations of T cells were finally analyzed on a flow cytometer. To analysis treatment-induced cytokine secretion, whole blood was collected from mice at 3 days post first treatment. The serum concentration of proinflammatory cytokines including TNF-α, IL-6 and IFN-γ were then analyzed with ELISA kit according to the manufacturer's instructions.

***In Vivo* Antimetastasis Effect**

The *in vivo* orthotopic breast tumor model was established by subcutaneously injecting 9 × 10^5^ 4T1 cells into the right mammary gland of Balb/c nude mice. When the tumor volume grew to about 200 mm^3^ after injection, the mice were randomly assigned to 6 different groups as mentioned above. Mice body weight and tumor volume in all groups were monitored every 3 days. A caliper was employed to measure the tumor length and tumor width, and the tumor volume was calculated according to following formula. Tumor volume = tumor length × tumor width^2^ / 2. After 15 days, the mice were sacrificed, and the lungs of each group were collected. The metastatic nodules on the pulmonary tissues were counted, and the lung sections were stained by hematoxylin and eosin (HE).

**Statistical analysis**

Data analyses were conducted using the GraphPad Prism 5.0 software. For variance analysis, One-way analysis of variance (ANOVA) with Tukey’s post hoc test was used. p values of <0.05 were considered significant. *p < 0.05, **p < 0.01, ***p < 0.001.

**References**

[1] Z. Chen, Q. Yang, P. Zhou, C. Li, X. Wang, B. Jiang, Q. Wang, Z. Xu, *Colloids and Surfaces A: Physicochemical and Engineering Aspects* **2024**, *702*.

[2] J. Liu, Q. You, S. Ye, J. Wang, R. Du, F. Liang, Z. He, C. Wang, L. Zhu, Y. Yang, *Nano Today* **2024**, *55*, 102148.

[3] J. Li, L. Xie, W. Sang, W. Li, G. Wang, J. Yan, Z. Zhang, H. Tian, Q. Fan, Y. Dai, *Angewandte Chemie International Edition* **2022**, *61*, e202200830.

[4] K. Ma, H. Cheng, L. Wang, H. Xiao, F. Liu, Y. Yang, Z. Xiao, K. Tang, S. Li, G. Wang, M. Ge, J. Wang, X. Liu, H.-X. Sun, Z. Luo, Z. Gu, P.-C. Ho, G. Li, L. Zhang, *Immunity* **2025**, *25*, 00326-00327.

**Supplementary figures**


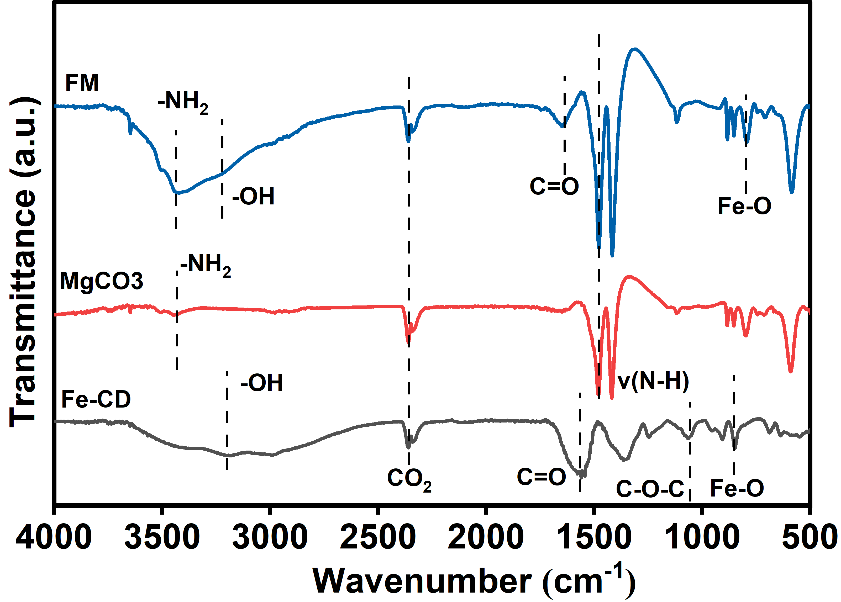


**Figure S1.** The Fourier Transform Infrared Spectroscopy‌ (FTIR) spectra of Fe-CD, MgCO_3_, and FM.


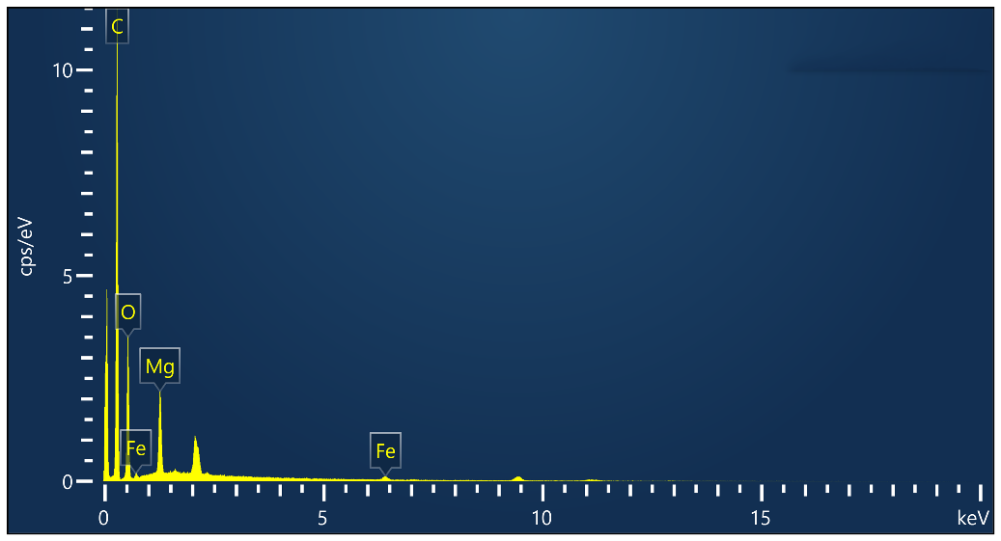


**Figure S2.** The Energy Dispersive Spectrometry (EDS) analysis of FM.


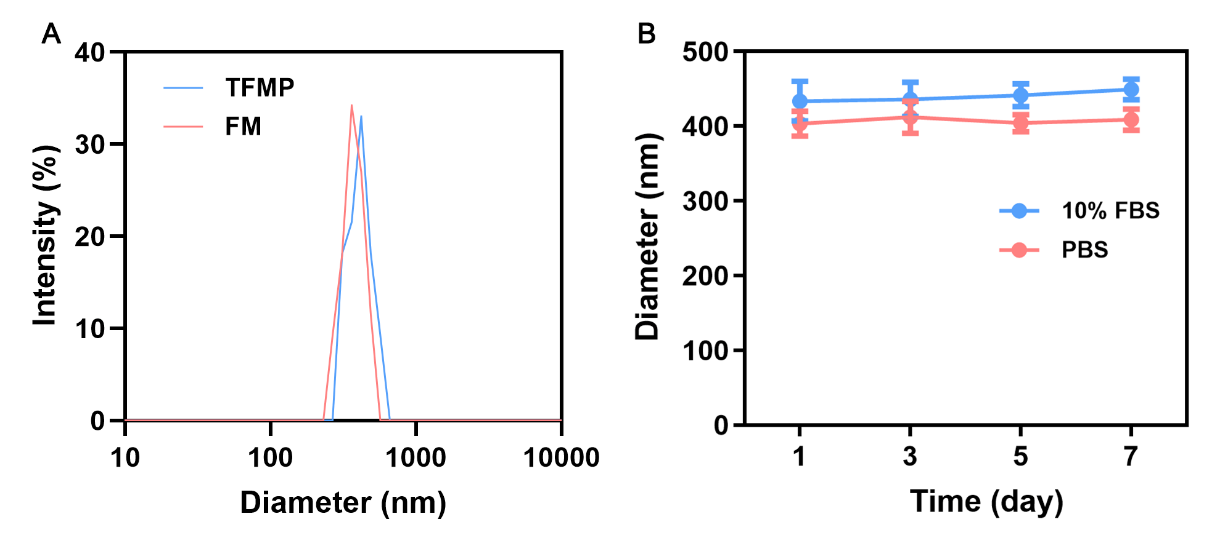


**Figure S3.** (A) The particle size distribution of different nanomaterials. (B) Stability of TFMP in various solutions at different times. Data are shown as the mean ± SD (n = 3).


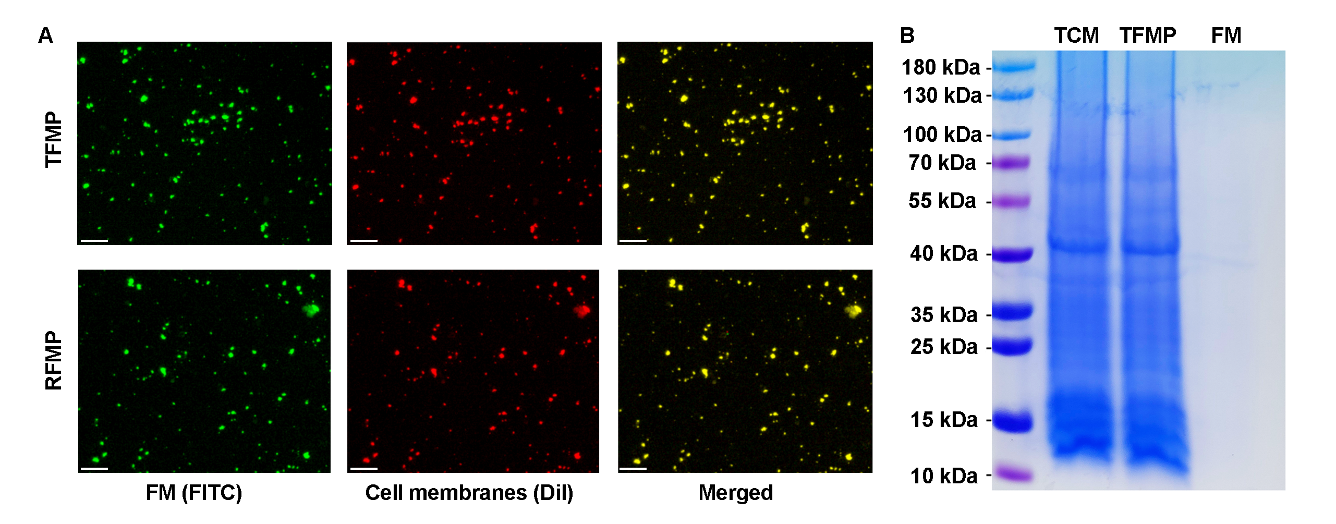


**Figure S4.** (A) Confocal microscopic analysis of the colocalization of FM (FITC) and cell membranes (Dil) within the TFMP or RFMP. Scale bars: 1μm. (B) The protein analysis of TCM, TFM, and FM by SDS-PAG.


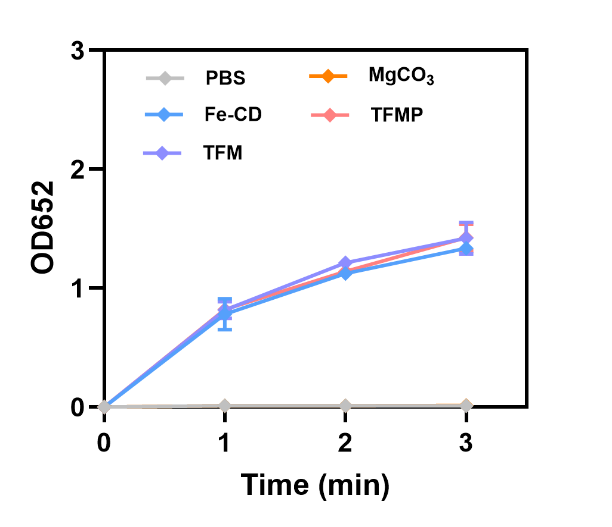


**Figure S5.** The OD values of oxTMB at 652 nm at different time points in different groups after co-incubation with TMB (5 μg/mL) and H_2_O_2_ (10 mM). Data are shown as the mean ± SD (n = 3).


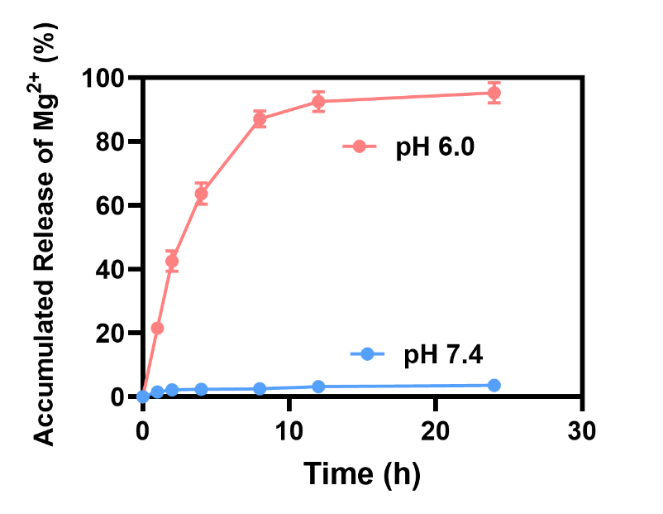


**Figure S6.** Mg^2+^ release profile under different pH value form TFMP. Data are shown as the mean ± SD (n = 3).


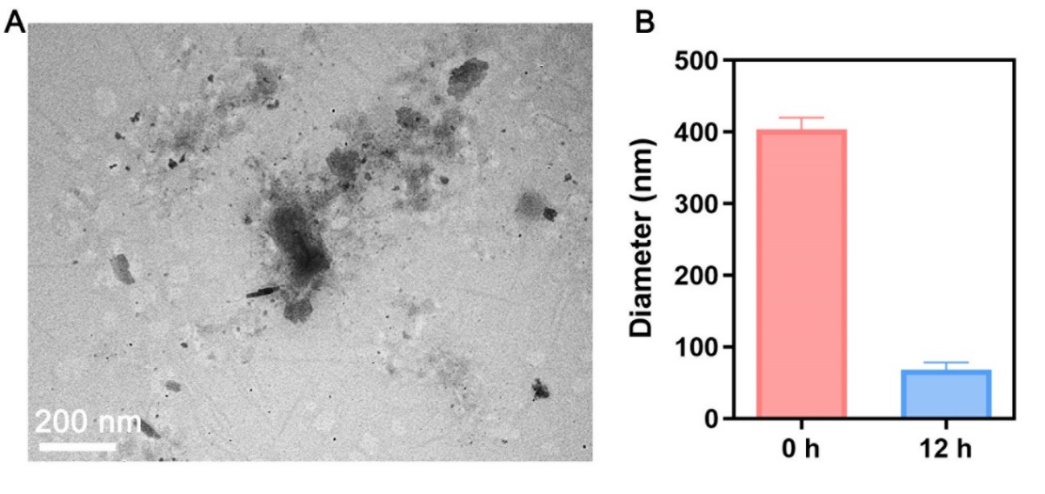


**Figure S7.** (A) TEM image of TFMP after incubation in a buffer solution with a pH of 6 for 12 hours. (B) The average particle size of TFMP before and after incubation in a buffer solution with a pH of 6 for 12 hours was determined by DLS.


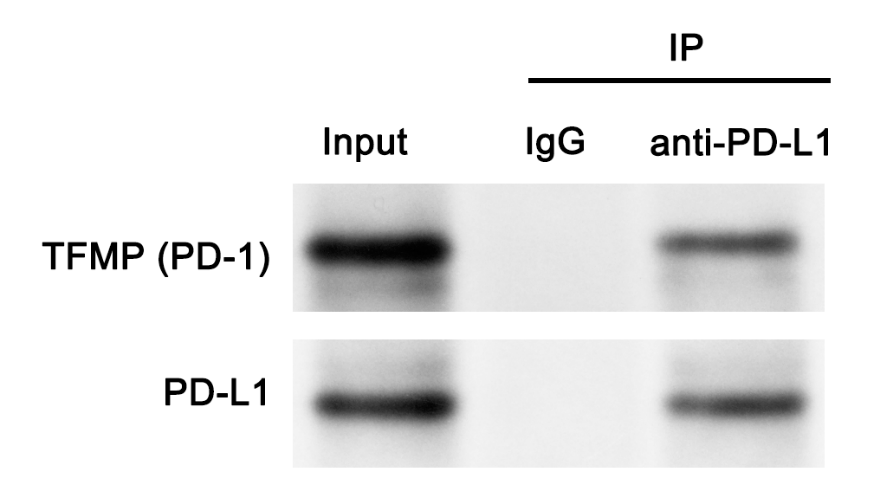


**Figure S8.** The Co-immunoprecipitation assay with anti-PD-L1 antibody (or IgG) showed the binding of TFMP to PD-L1, followed by immunoblotting of PD-1 and PD-L1.


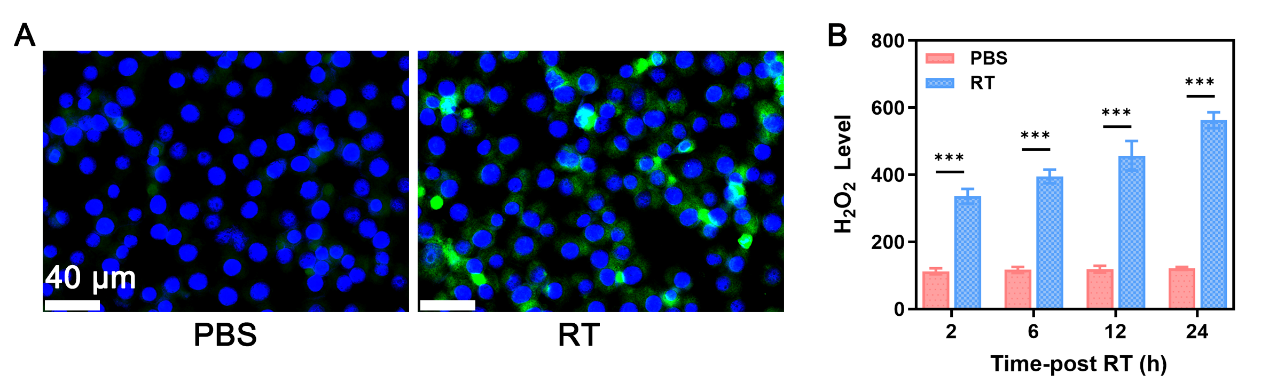


**Figure S9.** (A) DHE Fluorescence images of 4T1 cells after 4Gy-RT irradiation. (B) Production of intracellular H_2_O_2_ by RT. Data are shown as the mean ± SD (n = 3). Statistical significance was calculated via one-way ANOVA with Tukey’s test: **p < 0.01, ***p < 0.001.


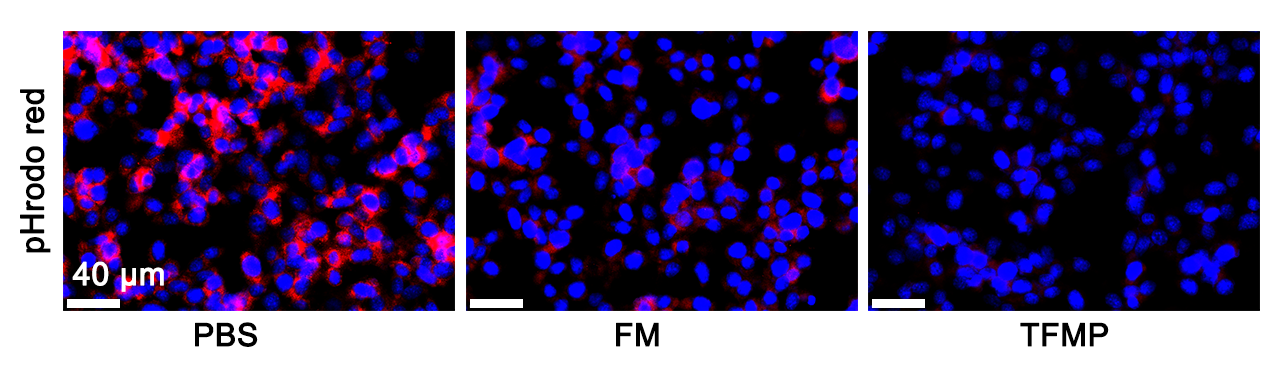


**Figure S10.** Fluorescence images of cancer cells showing detection of pHrodo red staining after the indicated treatments.


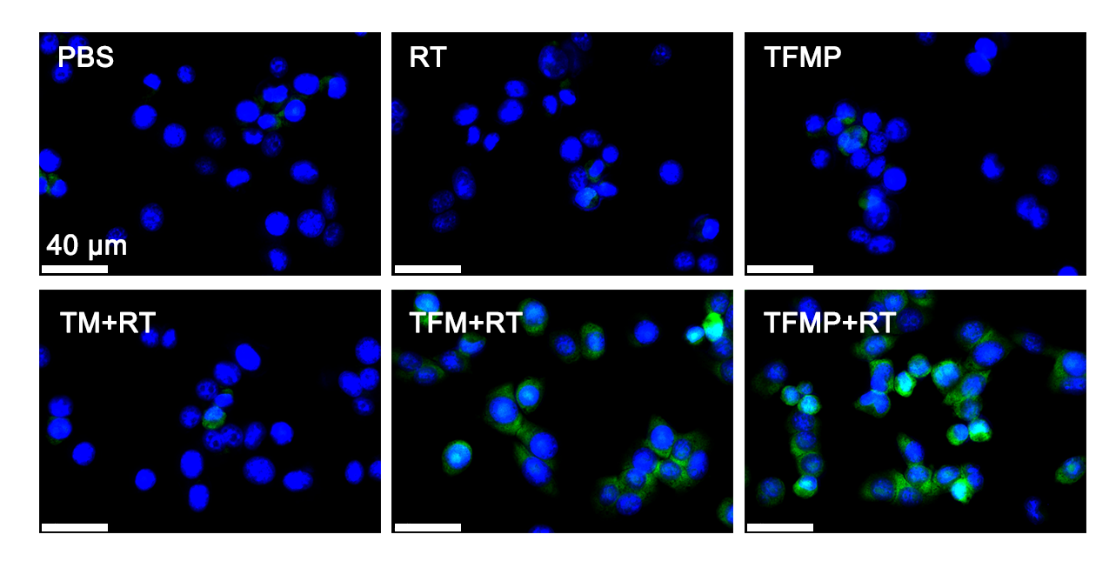


**Figure S11.** DCFH-DA fluorescence images of 4T1 cells after indicated treatments.


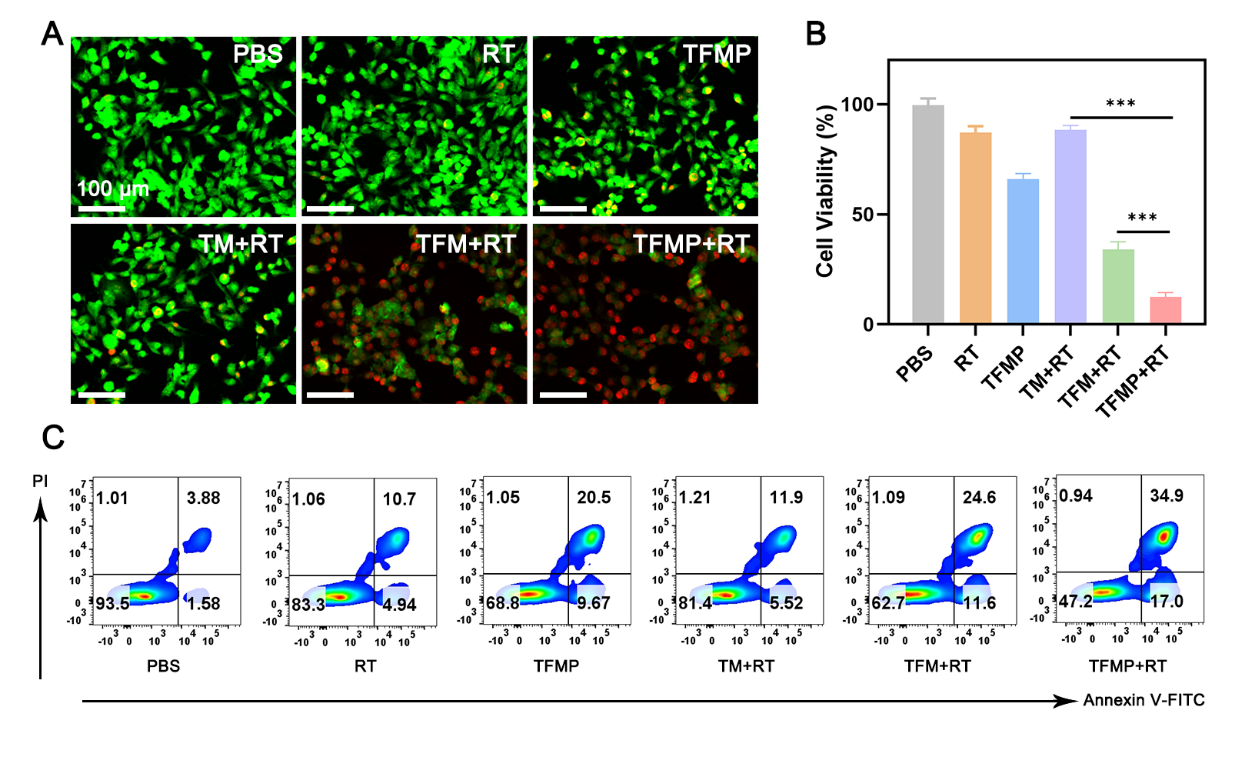


**Figure S12.** (A) Live/dead cell assay for cells treated with different conditions (green: live cells, red: dead cells). (B) Cell viability assay of 4T1 cells treated under different conditions. Data are shown as the mean ± SD (n = 3). (C) Flow cytometry analysis of the apoptosis of 4T1 cells incubated with different formulations after staining with Annexin V-FITC/PI. Statistical significance was calculated via one-way ANOVA with Tukey’s test: **p < 0.01, ***p < 0.001.


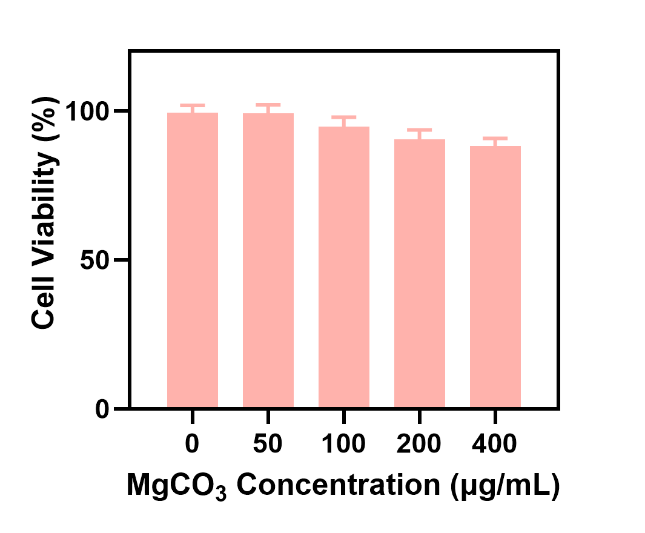


**Figure S13.** The cell viability of RAW 264.7 cells after TFMP treatments with different MgCO_3_ concentrations. Data are shown as the mean ± SD (n = 3).


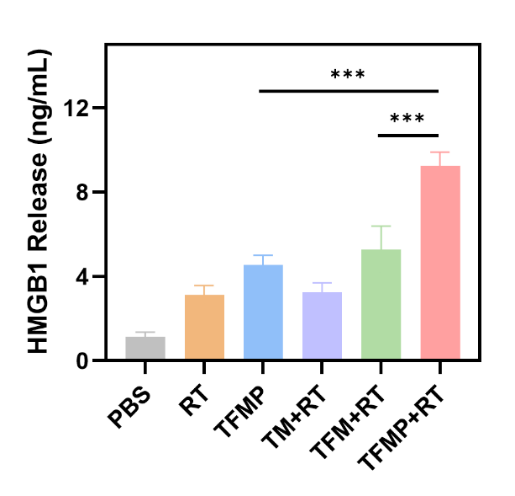


**Figure S14.** HMGB1 release from 4T1 cells after indicated treatments. Data are shown as the mean ± SD (n = 3). Statistical significance was calculated via one-way ANOVA with Tukey’s test: ***p < 0.001.


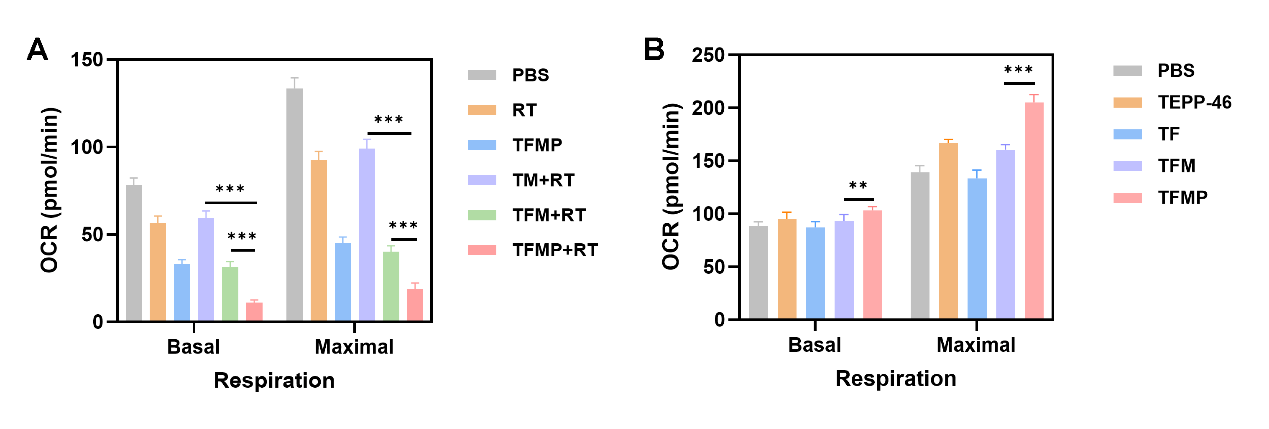


**Figure S15.** (A) Basal and maximal oxygen consumption rates (OCR) of 4T1 cells and (B) CD8^+^ T cells treated with indicated treatment. Data are shown as the mean ± SD (n = 3). Statistical significance was calculated via one-way ANOVA with Tukey’s test: **p < 0.01, ***p < 0.001.


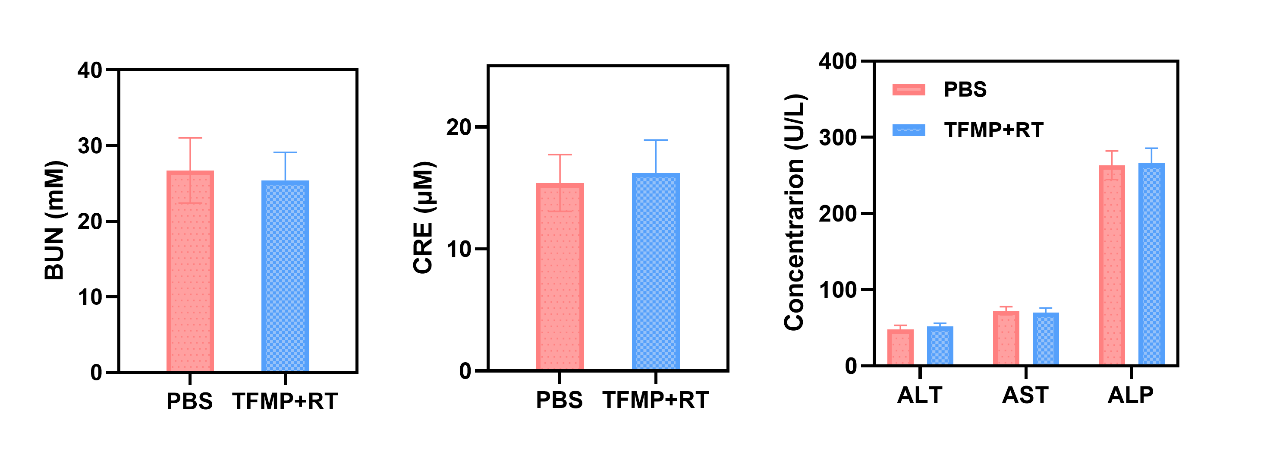


**Figure S16.** Analysis of liver and kidney function indicators. Data are shown as the mean ± SD (n = 5).


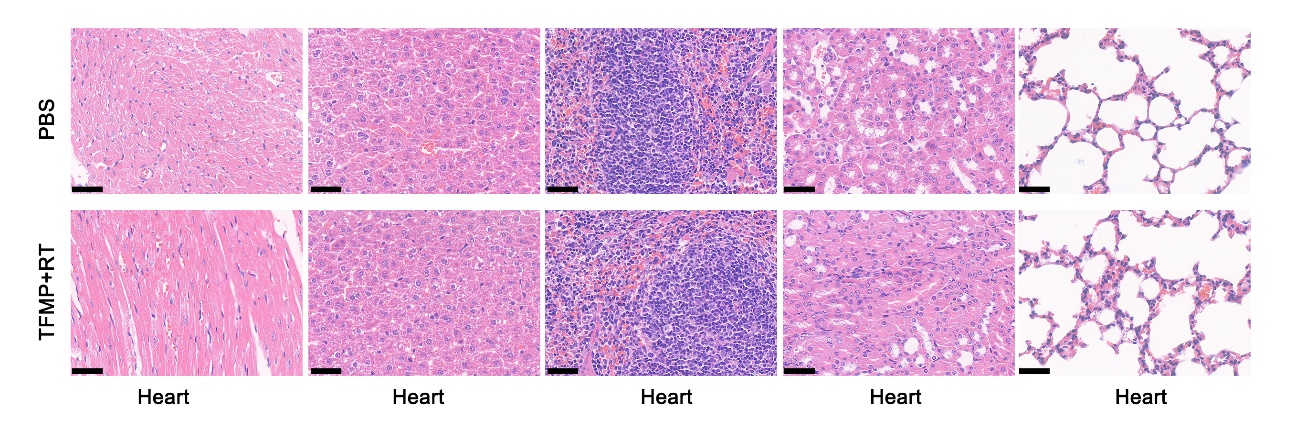


**Figure S17.** HE sections of major organs after different treatments. Scale bars: 40 μm.
